# Longitudinal Genome-Wide Association of Cardiovascular Disease Risk Factors in the Bogalusa Heart Study

**DOI:** 10.1371/journal.pgen.1001094

**Published:** 2010-09-09

**Authors:** Erin N. Smith, Wei Chen, Mika Kähönen, Johannes Kettunen, Terho Lehtimäki, Leena Peltonen, Olli T. Raitakari, Rany M. Salem, Nicholas J. Schork, Marian Shaw, Sathanur R. Srinivasan, Eric J. Topol, Jorma S. Viikari, Gerald S. Berenson, Sarah S. Murray

**Affiliations:** 1 Scripps Genomic Medicine and Scripps Translational Science Institute, La Jolla, California, United States of America; 2 Department of Epidemiology, Tulane University, New Orleans, Louisiana, United States of America; 3 Department of Clinical Physiology, University Hospital of Tampere and University of Tampere Medical School, Tampere, Finland; 4 Wellcome Trust Sanger Institute, Wellcome Trust Genome Campus, Hinxton, Cambridge, United Kingdom; 5 FIMM, Institute for Molecular Medicine Finland, Helsinki, Finland; 6 Department of Clinical Chemistry, University Hospital of Tampere and University of Tampere Medical School, Tampere, Finland; 7 The Broad Institute of MIT and Harvard, Boston, Massachusetts, United States of America; 8 Department of Clinical Physiology, Turku University Hospital and Centre of Applied and Preventive Cardiovascular Medicine, University of Turku, Turku, Finland; 9 Department of Medicine, University of Turku and Turku University Hospital, Turku, Finland; Georgia Institute of Technology, United States of America

## Abstract

Cardiovascular disease (CVD) is the leading cause of death worldwide. Recent genome-wide association (GWA) studies have pinpointed many loci associated with CVD risk factors in adults. It is unclear, however, if these loci predict trait levels at all ages, if they are associated with how a trait develops over time, or if they could be used to screen individuals who are pre-symptomatic to provide the opportunity for preventive measures before disease onset. We completed a genome-wide association study on participants in the longitudinal Bogalusa Heart Study (BHS) and have characterized the association between genetic factors and the development of CVD risk factors from childhood to adulthood. We report 7 genome-wide significant associations involving CVD risk factors, two of which have been previously reported. Top regions were tested for replication in the Young Finns Study (YF) and two associations strongly replicated: rs247616 in *CETP* with HDL levels (combined *P* = 9.7×10^−24^), and rs445925 at *APOE* with LDL levels (combined *P* = 8.7×10^−19^). We show that SNPs previously identified in adult cross-sectional studies tend to show age-independent effects in the BHS with effect sizes consistent with previous reports. Previously identified variants were associated with adult trait levels above and beyond those seen in childhood; however, variants with time-dependent effects were also promising predictors. This is the first GWA study to evaluate the role of common genetic variants in the development of CVD risk factors in children as they advance through adulthood and highlights the utility of using longitudinal studies to identify genetic predictors of adult traits in children.

## Author Summary

We have studied the association between genetic factors on a whole genome level and cardiovascular disease (CVD) risk factors in a population of individuals studied from childhood through adulthood. The longitudinal study design has enabled the investigation of genetic variation influencing trait values over time. We have identified DNA variants that are associated with CVD trait values consistently over time, and a second set of variants that are associated with CVD trait values in a time-dependent manner. We also show that variants previously identified in adult populations have consistent effects within our population and that these effects are usually similar across childhood through adulthood. The discovery of time-dependent variants that influence CVD trait values over time can potentially be used to screen young individuals who are pre-symptomatic and provide the opportunity for preventive measures decades before disease onset.

## Introduction

Cardiovascular disease (CVD) affects over 79 million people in the United States [1], and is the leading cause of death worldwide [2]–[4]. Identifying the genetic determinants of CVD can lead to more effective diagnostics, prognostics, therapeutics, and, ultimately, preventive strategies. The best chance for prevention would be to identify risk at the earliest possible age. Genome-wide association (GWA) leveraging cross-sectional phenotypic data has been a particularly useful approach to identifying loci that influence many of the quantitative risk factors of CVD [5]–[10], however the use of cross sectional data does not provide insight into how such risk factors develop over time. Longitudinal studies, particularly those that begin in childhood, allow for the identification of risk profiles of susceptible individuals before disease onset. The Bogalusa Heart Study (BHS) is a longitudinal study focused on the early natural history of CVD. The BHS began in 1973 and includes up to 9 phenotypic screenings in childhood (4–17 years of age) and up to 10 adult (18–48 years of age) cross-sectional screenings. We have conducted a longitudinal genome-wide association study on a subset of the total sample of unrelated individuals with a large number of measurements (mean number of measurements = 8, range = 4–13) and are of European Ancestry (N = 525).

## Results

### Longitudinal GWA

We conducted a genome-wide association study of longitudinal measures of 12 traits measured from childhood through adulthood on participants of the BHS of European ancestry: anthropomorphic (height, weight, and waist circumference), blood pressure (BP) (diastolic and systolic BP), heart rate, blood lipids (low density lipoprotein cholesterol (LDL), high density lipoprotein cholesterol (HDL), total cholesterol (TC), and triglycerides), and metabolic traits (glucose and insulin). Genotyping was performed on the Illumina Human610 and HumanCVD BeadChips [11] for a total of 545,821 SNPs passing QC and allele frequency filters (see Materials and Methods). Imputation was performed using the CEU HapMap 2 as a reference population with the computer program MACH v.1.0.16 (http://www.sph.umich.edu/csg/yli/mach/) [12], providing genotype estimates for an additional 1,622,114 SNPs. For each SNP, we tested whether it had an average linear effect over time (SNP effect), and whether it entered into a time-dependent effect (SNPxAGE interaction effect), such that the genotype is associated with variation in the linear trajectory of the trait from childhood through adulthood. Both SNP and SNPxAGE effects were calculated using linear mixed models as implemented in the R nlme package [13], adjusting for age and gender.

Table 1 lists all regions showing SNP effect associations (*P*<10^−6^) and Table 2 lists all regions showing association (*P*<10^−6^) with SNPxAGE effects. We analyzed the regions surrounding the top associations for consistency with recombination hotspots and LD relationships (Figure S1) and provide Manhattan plots of each trait association (Figure S2). From both sets of analyses, there were 5 novel associations with a *P*-value less than 5×10^−8^ and 6 novel regions where there were at least 10 genotyped or imputed SNPs with *P*<10^−5^. The most significant association (rs7890572, *P* = 3.8×10^−10^) was observed with a linear triglyceride trajectory effect (i.e., SNPxAGE effect) on the X chromosome within the *IL1RAPL1* gene and near the gene encoding glycerol kinase (*GK*), in which mutations have been implicated in pseudo-hypertriglyceridemia, caused by high levels of glycerol creating measurement artifacts in the triglyceride assay [14]. A novel association of potential biological interest involved a SNP effect on insulin levels with variation in the *CHN2* locus (rs3793275, *P* = 5.8×10^−9^), a beta-chimerin that has recently been described as part of a fusion gene also containing the insulin receptor that was shown to be responsible for severe insulin deficiency [15]. This SNP is also associated with glucose trajectories in our dataset (SNPxAGE; *P* = 1.5×10^−7^). In the 7q11 region, 25 SNPs are associated (*P*<10^−5^) with diastolic BP (SNP effect; peak SNP rs709595, *P* = 7.0×10^−7^). The calcitonin gene-related peptide receptor (*CRCP*) is approximately 200 kb from the top SNP, but contains SNPs that are in LD with the top SNP (see Figure S1). The calcitonin gene-related peptide is a vasodilator [16] and its receptor *CRCP* has been previously implicated in hypertension in a small candidate gene association study of hypertension in Japanese individuals [17].

**Table 1:** Top SNP effect GWA hits for 12 phenotypes.

Trait	Cytoband	Gene(s)	Top SNP, Alleles	#SNPs @ *P*<10^−5^	Risk AF	Beta (SE)	*P*
diastolic BP	5p13.3	*CDH9/CDH6*	rs7704530 G/A	*2/3*	0.26	8.44 (1.57)	1.1×10^−7^
diastolic BP	7q11.21	*TPST1*	rs709595 C/G	*8/17*	0.39	7.18 (1.43)	7.0×10^−7^
glucose	2q24.3	*G6PC2, ABCB11*	rs853773 A/G	*4/1*	0.53	−8.72 (1.73)	7.0×10^−7^
glucose	6q22.31	*NKAIN2/RNF217*	rs781718* G/A	*1/4*	0.89	−3.46 (0.69)	8.4×10^−7^
HDL-cholesterol	16q12.2	*HERPUD1/CETP*	rs247616* T/C	*4/2*	0.33	3.77 (0.75)	6.6×10^−7^
**insulin**	**7p14.3**	***CHN2***	rs3793275* A/T	***1/3***	**0.94**	−4.63 (0.78)	**5.8×10^−9^**
insulin	20p13	*RNF24/SMOX*	rs6052399* T/C	*0/1*	0.93	−4.55 (0.84)	9.8×10^−8^
insulin	6q14.1	*BCKDHB/FAM46A*	rs16892328* C/T	*0/1*	0.94	−4.93 (0.92)	1.3×10^−7^
insulin	18p11.31	*MRLC2/TGIF1*	rs1613695* G/A	*0/1*	0.95	−5.68 (1.07)	1.7×10^−7^
insulin	14q13.3	*TTC6/SSTR1*	rs10498337 T/G	*1/0*	0.81	−9.3 (1.87)	8.9×10^−7^
**LDL-cholesterol**	**21q22.11**	***MRPS6/KCNE2***	rs8131349* A/G	***0/1***	**0.06**	17.6 (3.05)	**1.4×10^−8^**
**LDL-cholesterol**	**19q13.33**	***APOE/APOC1***	**rs7412 T/C**	***1/1***	**0.07**	−69.74 (12.15)	**1.6×10^−8^**
LDL-cholesterol	6q22.31	*C6orf170/GJA1*	rs7738656 G/A	*1/0*	0.84	−42.39 (8.11)	2.5×10^−7^
LDL-cholesterol	5q33.2	*KIF4B/SGCD*	rs10044666* G/T	*1/3*	0.72	−8.57 (1.68)	4.7×10^−7^
systolic BP	11q24.1	*ASAM/GRAMD1B*	rs11822822 G/A	*2/4*	0.24	10.74 (2.12)	5.6×10^−7^
systolic BP	15q22.1	*RORA/VPS13C*	rs726914 G/A	*2/0*	0.62	9.21 (1.83)	7.1×10^−7^
**total cholesterol**	**21q22.11**	***MRPS6/KCNE2***	rs8131349* A/G	***2/2***	**0.06**	18.4 (3.32)	**4.6×10^−8^**
total cholesterol	4p16.1	*ABLIM2*	rs6829649 T/G	*1/2*	0.87	−50.96 (9.42)	9.6×10^−8^
total cholesterol	6q22.31	*C6orf170/GJA1*	rs7738656 G/A	*1/1*	0.84	−46.21 (8.79)	2.1×10^−7^
total cholesterol	8q24.22	*ST3GAL1/ZFAT*	rs4897695* C/G	*1/6*	0.91	13.66 (2.62)	2.7×10^−7^
waist circumference	8q24.13	*MTSS1/ZNF572*	rs891541* A/G	*4/7*	0.29	4.55 (0.91)	7.7×10^−7^

A list of all SNP effect *P*-values less than 10^−6^ in the BHS. SNP names marked with an “*” are imputed, while those that are unmarked are directly genotyped. SNP alleles are reported as risk/nonrisk and are in genome forward orientation (build 36.3). The number of SNPs @ *P*<10^−5^ corresponds to the number of genotyped/imputed SNPs with *P*<10^−5^ within 200kb up and downstream of the top SNP. Associations at *P*<5×10^−8^ are indicated in bold.

**Table 2:** Top SNPxAGE effect GWA hits for 12 phenotypes.

Trait	Cytoband	Gene(s)	Top SNP, Alleles	#SNPs @ *P*<10−5	Risk AF	Beta (SE)	*P*
distolic BP	14q24.3	*ESRRB/VASH1*	rs17104804 G/A	1/2	0.92	−0.76 (0.15)	5.9×10^−7^
glucose	16q21	*CDH5/TK2*	rs4783595 T/C	2/1	0.87	−1.67 (0.31)	9.5×10^−8^
glucose	7p14.3	*CHN2*	rs3793275* A/T	1/3	0.94	−0.61 (0.12)	1.5×10^−7^
glucose	Xp22.2	*ATXN3L/EGFL6*	rs5979903 T/C	1/0	0.06	1.88 (0.36)	1.9×10^−7^
glucose	11q13.5	*WNT11/PRKRIR*	rs12807555* G/T	0/1	0.90	−0.47 (0.09)	2.7×10^−7^
glucose	17q25.1	*PRPSAP1/SPHK1*	rs9909931 G/A	2/3	0.19	1.36 (0.27)	4.3×10^−7^
glucose	1q32.1	*PLXNA2/LOC642587*	rs12069004* C/T	0/1	0.91	−0.54 (0.11)	5.8×10^−7^
glucose	8q24.22	*ZFAT/KHDRBS3*	rs12548494* C/G	0/1	0.93	−0.6 (0.12)	7.1×10^−7^
glucose	7p21.1	*AHR/SNX13*	rs10260737 G/A	2/12	0.89	−1.77 (0.36)	7.3×10^−7^
glucose	8q23.2	*KCNV1/CSMD3*	rs3019325* G/C	0/6	0.70	−0.31 (0.06)	7.5×10^−7^
heart rate	3q24	*RASA2/RNF7*	rs6440031 G/A	1/0	0.90	1.33 (0.25)	1.8×10^−7^
heart rate	12q21.2	*NAV3/SYT1*	rs1449460 G/A	2/6	0.08	−1.36 (0.27)	7.4×10^−7^
height	11q23.3	*CADM1/BUD13*	rs1144036 C/T	1/0	0.16	1.17 (0.23)	2.4×10^−7^
height	19q13.32	*PSG3*	rs8103264* C/G	2/10	0.93	−0.38 (0.08)	5.6×10^−7^
insulin	12p11.23	*TM7SF3*	rs1552257* C/T	4/10	0.20	0.18 (0.04)	7.5×10^−7^
LDL	17q22	*AKAP1/MSI2*	rs8073909 T/C	1/0	0.64	−1.41 (0.27)	1.4×10^−7^
LDL	10p13	*FRMD4A*	rs11258628* A/T	0/1	0.92	−0.69 (0.14)	4.1×10^−7^
systolic BP	19p13.3	*RFX2*	rs1046391* G/T	0/1	0.86	0.21 (0.04)	4.1×10^−7^
systolic BP	14q24.3	*ESRRB/VASH1*	rs17104804 G/A	3/5	0.92	−0.91 (0.19)	9.3×10^−7^
total cholesterol	17q22	*AKAP1/MSI2*	rs8073909 T/C	1/4	0.64	−1.68 (0.31)	6.9×10^−8^
**triglycerides**	**Xp21.2**	***IL1RAPL1***	**rs7890572 G/A**	**5/0**	**0.07**	9.75 (1.55)	**3.8×10^−10^**
**triglycerides**	**11q23.3**	***CADM1/BUD13***	rs12280753* T/C	**7/11**	**0.07**	2.64 (0.47)	**1.8×10^−8^**
triglycerides	9q21.2	*PSAT1/CHCHD9*	rs13290397* C/G	1/3	0.89	−1.98 (0.38)	2.4×10^−7^
triglycerides	2p16.3	*FSHR/NRXN1*	rs6726786* G/T	1/3	0.89	−2.09 (0.41)	2.9×10^−7^
triglycerides	3q22.3	*IL20RB/SOX14*	rs12330441 T/G	2/4	0.93	−9.54 (1.86)	3.0×10^−7^
**waist circumference**	**20q13.32**	***APCDD1L/STX16***	rs127430* G/A	**2/13**	**0.86**	−0.33 (0.06)	**3.3×10^−8^**
waist circumference	11q24.1	*SORL1/BLID*	rs7121446 G/A	1/7	0.78	−1.09 (0.21)	3.6×10^−7^
waist circumference	17p13.2	*SHPK*	rs7210277* C/T	0/1	0.91	−0.39 (0.08)	7.0×10^−7^
waist circumference	10q24.2	*CNNM1*	rs17568778 C/A	1/0	0.92	−1.55 (0.31)	8.2×10^−7^
weight	4q35.1	*ODZ3*	rs6552560 T/C	3/7	0.23	1.35 (0.25)	8.5×10^−8^

A list of all SNPxAGE effect P-values less than 10^−6^ in the BHS. Associations at *P*<5×10^−8^ are indicated in bold. SNP names marked with an “*” are imputed, while those that are unmarked are not. SNP alleles are reported as risk/nonrisk and are in genome forward orientaiton (build 36.3). The number of SNPs at *P*<10^−5^ corresponds to the number of genotyped/imputed SNPs with *P*<10^−5^ within 200kb up and downstream of the top SNP.

In addition to novel associations, there were three regions showing SNP associations that have been previously identified in GWA studies: rs853773 [18] near *G6PC2* was associated with a glucose SNP effect (*P* = 7.0×10^−7^), rs247616 [5] near *CETP* was associated with an HDL SNP effect (*P* = 6.6×10^−7^), and the APOE e2 SNP rs7412 [19] was associated with a genome-wide significant LDL SNP effect (*P* = 1.6×10^−8^). A region near *APOA5* that had been previously implicated in triglyceride levels showed a significant SNPxAGE effect on triglycerides in our study (rs12280753; *P* = 1.8×10^−8^). Although the nearest gene to rs12280753 is not *APOA5*, this SNP was also the most strongly associated SNP in previous studies of adult triglyceride levels [5], [10], [20].

### Replication in the Young Finns

We pursued replication of these findings in genotyped individuals within the Young Finns (YF) cohort, consisting of 2,442 Finnish individuals tracked from childhood through middle adulthood (ages 3–45) with three measures in young individuals (ages 3–24) and two measures in older individuals (ages 24–45). These individuals have been genotyped on a custom-built Illumina genotyping chip (670K). Using the same analysis methods, we tested whether the top SNP was associated in the YF study (Table 3). Imputed genotype dosages were used when direct genotype data was not available. For the APOE-e2 SNP rs7412, which is not in HapMap or on the 670K chip, we used the SNP with the next strongest association in the BHS (rs445925). There were two SNPs that significantly replicated beyond the multiple testing threshold (*P*<0.05/51 = 1×10^−3^): the rs247616 SNP at *CETP* (*P* = 1.7×10^−18^), and rs445925 at *APOE* (*P* = 4.1×10^−15^). There was no trend to replicate the direction of effect between the studies: within the SNP effects, there were 12/21 (57%, chi-square *P* = 0.51) markers that showed the same direction of effect, while within SNPxAGE effects, there were 14/30 (47%, chi-square *P* = 0.72). The samples were combined and *P*-values were calculated for the combined BHS and YF data, using study as a covariate (Table 3). The associations at rs247616 at *CETP* with HDL-cholesterol (*P* = 9.7×10^−24^) and rs445925 at *APOE* with LDL-cholesterol (*P* = 8.7×10^−19^) were strongly significant, but no other regions in the combined BHS/YF data reached genome-wide significance of *P*<5×10^−8^.

**Table 3:** Replication results in the Young Finns.

			BHS		YF		BHS + YF combined	
Trait	Top SNP (Alleles)	Effect	Beta (SE)	*P*	Beta(SE)	*P*	Beta(SE)	*P*
diastolic BP	rs7704530 G/A	SNP	8.44 (1.57)	1.1×10^−7^	−0.05 (0.22)	0.82	0.4 (0.2)	0.04
diastolic BP	rs709595 C/G	SNP	7.18 (1.43)	7.0×10^−7^	−0.03 (0.21)	0.88	0.38 (0.19)	0.04
glucose	rs853773 A/G	SNP	−8.72 (1.73)	7.0×10^−7^	−0.33 (0.37)	0.37	−0.73 (0.32)	0.02
glucose	rs781718* G/A	SNP	−3.46 (0.69)	8.4×10^−7^	0.71 (0.7)	0.31	−0.32 (0.59)	0.58
**HDL-cholesterol**	rs247616* T/C	**SNP**	**3.77 (0.75)**	**6.6×10^−7^**	**2.8 (0.32)**	**1.7×10^−18^**	**2.99 (0.29)**	**9.7×10^−24^**
**insulin**	rs3793275* A/T	**SNP**	**−4.63 (0.78)**	**5.8×10^−9^**	**0.36 (0.3)**	**0.23**	**−0.13 (0.26)**	**0.63**
insulin	rs6052399* T/C	SNP	−4.55 (0.84)	9.8×10^−8^	−0.01 (0.44)	0.99	−0.7 (0.37)	0.06
insulin	rs16892328* C/T	SNP	−4.93 (0.92)	1.3×10^−7^	−0.81 (0.54)	0.14	−1.64 (0.45)	0.00027
insulin	rs1613695* G/A	SNP	−5.68 (1.07)	1.7×10^−7^	0.35 (0.36)	0.32	−0.13 (0.31)	0.68
insulin	rs10498337 T/G	SNP	−9.3 (1.87)	8.9×10^−7^	−0.05 (0.22)	0.81	−0.35 (0.18)	0.06
**LDL-cholesterol**	rs8131349* A/G	**SNP**	**17.6 (3.05)**	**1.4×10^−8^**	**−1.14 (1.29)**	**0.38**	**1.46 (1.2)**	**0.23**
**LDL-cholesterol**	rs445925*# G/A	**SNP**	**12.8 (2.63)**	**1.5×10^−6^**	**12.16 (1.54)**	**4.1×10^−15^**	**11.96 (1.34)**	**8.7×10^−19^**
LDL-cholesterol	rs7738656 G/A	SNP	−42.39 (8.11)	2.5×10^−7^	−1.52 (0.9)	0.09	−2.91 (0.83)	0.00049
LDL-cholesterol	rs10044666* G/T	SNP	−8.57 (1.68)	4.7×10^−7^	−1.25 (0.91)	0.17	−2.93 (0.81)	0.00029
systolic BP	rs11822822 G/A	SNP	10.74 (2.12)	5.6×10^−7^	−0.34 (0.35)	0.34	0.44 (0.3)	0.14
systolic BP	rs726914 G/A	SNP	9.21 (1.83)	7.1×10^−7^	0.07 (0.27)	0.81	0.55 (0.23)	0.02
**total cholesterol**	rs8131349* A/G	**SNP**	**18.4 (3.32)**	**4.6×10^−8^**	**−1.26 (1.43)**	**0.38**	**1.44 (1.32)**	**0.28**
total cholesterol	rs6829649 T/G	SNP	−50.96 (9.42)	9.6×10^−8^	0.09 (1.23)	0.94	−2.06 (1.1)	0.06
total cholesterol	rs7738656 G/A	SNP	−46.21 (8.79)	2.1×10^−7^	−1.51 (1)	0.13	−3.04 (0.92)	0.00094
total cholesterol	rs4897695* C/G	SNP	13.66 (2.62)	2.7×10^−7^	0.51 (1.7)	0.76	4.14 (1.44)	0.0041
waist circumference	rs891541* A/G	SNP	4.55 (0.91)	7.7×10^−7^	0.28 (0.37)	0.44	1.18 (0.35)	0.00076
distolic BP	rs17104804 G/A	SNPxAGE	−0.76 (0.15)	5.9×10^−7^	−0.02 (0.04)	0.63	−0.1 (0.03)	0.00047
glucose	rs4783595 T/C	SNPxAGE	−1.67 (0.31)	9.5×10^−8^	−0.04 (0.04)	0.29	−0.12 (0.03)	0.00021
glucose	rs3793275* A/T	SNPxAGE	−0.61 (0.12)	1.5×10^−7^	0.04 (0.05)	0.43	−0.09 (0.05)	0.05
glucose	rs5979903 T/C	SNPxAGE	1.88 (0.36)	1.9×10^−7^	0.08 (0.04)	0.07	0.16 (0.04)	9.8×10^−5^
glucose	rs12807555* G/T	SNPxAGE	−0.47 (0.09)	2.7×10^−7^	0.01 (0.05)	0.78	−0.1 (0.04)	0.02
glucose	rs9909931 G/A	SNPxAGE	1.36 (0.27)	4.3×10^−7^	−0.02 (0.04)	0.56	0.06 (0.03)	0.06
glucose	rs12069004* C/T	SNPxAGE	−0.54 (0.11)	5.8×10^−7^	0.02 (0.05)	0.7	−0.1 (0.05)	0.03
glucose	rs12548494* C/G	SNPxAGE	−0.6 (0.12)	7.1×10^−7^	1.4×10−4 (0.09)	1	−0.18 (0.07)	0.01
glucose	rs10260737 G/A	SNPxAGE	−1.77 (0.36)	7.3×10^−7^	−0.02 (0.06)	0.8	−0.11 (0.05)	0.03
glucose	rs3019325* G/C	SNPxAGE	−0.31 (0.06)	7.5×10^−7^	−0.02 (0.03)	0.55	−0.07 (0.03)	0.0097
heart rate	rs6440031 G/A	SNPxAGE	1.33 (0.25)	1.8×10^−7^	−0.01 (0.03)	0.72	0.03 (0.02)	0.16
heart rate	rs1449460 G/A	SNPxAGE	−1.36 (0.27)	7.4×10^−7^	0 (0.03)	0.93	−0.05 (0.03)	0.1
height	rs1144036 C/T	SNPxAGE	1.17 (0.23)	2.4×10^−7^	−0.07 (0.04)	0.09	0 (0.03)	0.91
height	rs8103264* C/G	SNPxAGE	−0.38 (0.08)	5.6×10^−7^	−0.04 (0.06)	0.42	−0.11 (0.05)	0.02
insulin	rs1552257* C/T	SNPxAGE	0.18 (0.04)	7.5×10^−7^	0 (0.02)	0.79	0.02 (0.02)	0.27
LDL	rs8073909 T/C	SNPxAGE	−1.41 (0.27)	1.4×10^−7^	0.06 (0.04)	0.08	0.04 (0.04)	0.32
LDL	rs11258628* A/T	SNPxAGE	−0.69 (0.14)	4.1×10^−7^	0.02 (0.09)	0.84	−0.38 (0.09)	1.8×10^−5^
systolic BP	rs1046391* G/T	SNPxAGE	0.21 (0.04)	4.1×10^−7^	0.01 (0.03)	0.78	0.05 (0.02)	0.03
systolic BP	rs17104804 G/A	SNPxAGE	−0.91 (0.19)	9.3×10^−7^	0.01 (0.03)	0.63	−0.05 (0.03)	0.06
total cholesterol	rs8073909 T/C	SNPxAGE	−1.68 (0.31)	6.9×10^−8^	0.06 (0.04)	0.13	0.03 (0.04)	0.51
**triglycerides**	**rs7890572 G/A**	**SNPxAGE**	**9.75 (1.55)**	**3.8×10^−10^**	**0.1 (0.22)**	**0.66**	**0.97 (0.18)**	**1.2×10^−7^**
**triglycerides**	rs12280753* T/C	**SNPxAGE**	**2.64 (0.47)**	**1.8×10^−8^**	**0.28 (0.16)**	**0.09**	**0.69 (0.15)**	**7.8×10^−6^**
triglycerides	rs13290397* C/G	SNPxAGE	−1.98 (0.38)	2.4×10^−7^	0.21 (0.15)	0.17	−0.25 (0.14)	0.08
triglycerides	rs6726786* G/T	SNPxAGE	−2.09 (0.41)	2.9×10^−7^	0.1 (0.15)	0.49	−0.32 (0.14)	0.02
triglycerides	rs12330441 T/G	SNPxAGE	−9.54 (1.86)	3.0×10^−7^	−0.05 (0.16)	0.75	−0.47 (0.16)	0.0025
**waist circumference**	rs127430* G/A	**SNPxAGE**	**−0.33 (0.06)**	**3.3×10^−8^**	**−0.02 (0.05)**	**0.65**	**−0.19 (0.04)**	**1.4×10^−6^**
waist circumference	rs7121446 G/A	SNPxAGE	−1.09 (0.21)	3.6×10^−7^	−0.07 (0.04)	0.11	−0.15 (0.03)	2.5×10^−6^
waist circumference	rs7210277* C/T	SNPxAGE	−0.39 (0.08)	7.0×10^−7^	0.04 (0.06)	0.48	−0.17 (0.05)	0.00043
waist circumference	rs17568778 C/A	SNPxAGE	−1.55 (0.31)	8.2×10^−7^	0 (0.06)	0.98	−0.17 (0.05)	0.00024
weight	rs6552560 T/C	SNPxAGE	1.35 (0.25)	8.5×10^−8^	−0.02 (0.03)	0.5	0.06 (0.03)	0.03

Replication effects and P-values in the Young Finns (YF) Study and in combined data, adjusted for study. SNP names marked with an “*” are imputed, while those that are unmarked were directly genotyped. SNP alleles are reported as risk/nonrisk and are in genome forward orientation (build 36.3). # rs445925 is a proxy for rs7412.

### Prediction of adult values given childhood values

Genetic variants will be most useful for trait prediction when they are associated with a trait above and beyond other known risk factors. In addition, the ability to predict adult trait levels in children, before disease onset, can lead to a disease prevention strategy. In longitudinal studies starting in childhood and going into adulthood, we can ask whether genetic loci are associated with the adult trait level above and beyond the trait level seen in the first measure taken in childhood. To test this hypothesis, we evaluated whether our associated markers were likely to be predictive of adult levels of the traits, after adjustment for trait levels in childhood. To account for variation in data collection, we also included the age at each of these measures as well as gender as covariates in the analysis. Within the BHS, variants that were characterized as SNPxAGE effects were more likely to be predictive of adult values after correcting for childhood values, which is expected since these variants were characterized in BHS initially (Table 4). In the YF study, however, we also saw more SNPxAGE variants associated with adult levels given childhood levels (Table 4). There were 6 variants that were associated with adult levels in the YF study at *P*<0.05, with 2 corresponding to the genome-wide significant SNP effects and 4 corresponding to BHS SNPxAGE variants. Only the association of rs445925 with LDL-cholesterol was strong enough to withstand multiple corrections. Further analysis of this observation is warranted in a larger cohort.

**Table 4:** Association of GWAS SNPs with adult trait levels after adjusting for childhood levels.

Trait	Top SNP (Alleles)	Effect	BHS Adult prediction *P*	YF Adult prediction *P*
diastolic BP	rs7704530 G/A	SNP	0.07	0.74
diastolic BP	rs709595 C/G	SNP	0.26	0.09
glucose	rs853773 A/G	SNP	0.003	0.24
glucose	rs781718* G/A	SNP	0.27	0.21
HDL-cholesterol	rs247616* T/C	SNP	8.1×10^−4^	0.0067
insulin	rs3793275* A/T	SNP	0.89	0.36
insulin	rs6052399* T/C	SNP	0.014	0.89
insulin	rs16892328* C/T	SNP	0.22	0.14
insulin	rs1613695* G/A	SNP	0.16	0.67
insulin	rs10498337 T/G	SNP	0.38	0.88
LDL-cholesterol	rs8131349* A/G	SNP	0.14	0.16
LDL-cholesterol	rs445925* G/A	SNP	0.025	2.3×10^−6^
LDL-cholesterol	rs7738656 G/A	SNP	0.042	0.25
LDL-cholesterol	rs10044666* G/T	SNP	0.015	0.78
systolic BP	rs11822822 G/A	SNP	0.002	0.63
systolic BP	rs726914 G/A	SNP	0.004	0.54
total cholesterol	rs8131349* A/G	SNP	0.051	0.3
total cholesterol	rs6829649 T/G	SNP	0.56	0.44
total cholesterol	rs7738656 G/A	SNP	0.028	0.08
total cholesterol	rs4897695* C/G	SNP	0.24	0.91
waist circumference	rs891541* A/G	SNP	0.14	0.28
distolic BP	rs17104804 G/A	SNPxAGE	1.5×10^−4^	0.46
glucose	rs4783595 T/C	SNPxAGE	8.7×10^−8^	0.4
glucose	rs3793275* A/T	SNPxAGE	5.7×10^−7^	0.44
glucose	rs5979903 T/C	SNPxAGE	3.0×10^−4^	0.45
glucose	rs12807555* G/T	SNPxAGE	1.2×10^−8^	0.93
glucose	rs9909931 G/A	SNPxAGE	5.0×10^−7^	0.67
glucose	rs12069004* C/T	SNPxAGE	3.2×10^−5^	0.88
glucose	rs12548494* C/G	SNPxAGE	2.7×10^−6^	0.45
glucose	rs10260737 G/A	SNPxAGE	1.2×10^−5^	1
glucose	rs3019325* G/C	SNPxAGE	2.2×10^−5^	0.56
heart rate	rs6440031 G/A	SNPxAGE	1.2×10^−3^	0.77
heart rate	rs1449460 G/A	SNPxAGE	0.0302	0.81
height	rs1144036 C/T	SNPxAGE	0.2199	0.01
height	rs8103264* C/G	SNPxAGE	0.0360	0.49
insulin	rs1552257* C/T	SNPxAGE	3.8×10^−5^	0.61
LDL	rs8073909 T/C	SNPxAGE	1.2×10^−6^	0.007
LDL	rs11258628* A/T	SNPxAGE	7.3×10^−6^	0.88
systolic BP	rs1046391* G/T	SNPxAGE	6.2×10^−4^	0.74
systolic BP	rs17104804 G/A	SNPxAGE	0.0015	0.36
total cholesterol	rs8073909 T/C	SNPxAGE	2.0×10^−7^	0.008
triglycerides	rs7890572 G/A	SNPxAGE	4.2×10^−6^	0.43
triglycerides	rs12280753* T/C	SNPxAGE	9.9×10^−9^	0.24
triglycerides	rs13290397* C/G	SNPxAGE	6.8×10^−7^	0.33
triglycerides	rs6726786* G/T	SNPxAGE	3.9×10^−6^	0.74
triglycerides	rs12330441 T/G	SNPxAGE	3.1×10^−5^	0.79
waist circumference	rs127430* G/A	SNPxAGE	2.1×10^−7^	0.71
waist circumference	rs7121446 G/A	SNPxAGE	2.2×10^−6^	0.68
waist circumference	rs7210277* C/T	SNPxAGE	1.2×10^−6^	0.9
waist circumference	rs17568778 C/A	SNPxAGE	9.0×10^−7^	0.99
weight	rs6552560 T/C	SNPxAGE	1.8×10^−5^	0.04

Adult prediction *P*-values correspond to the association between the SNP and the last adult measurement, after adjusting for the first measure in childhood.

### Previously identified markers

**Figure 1:**
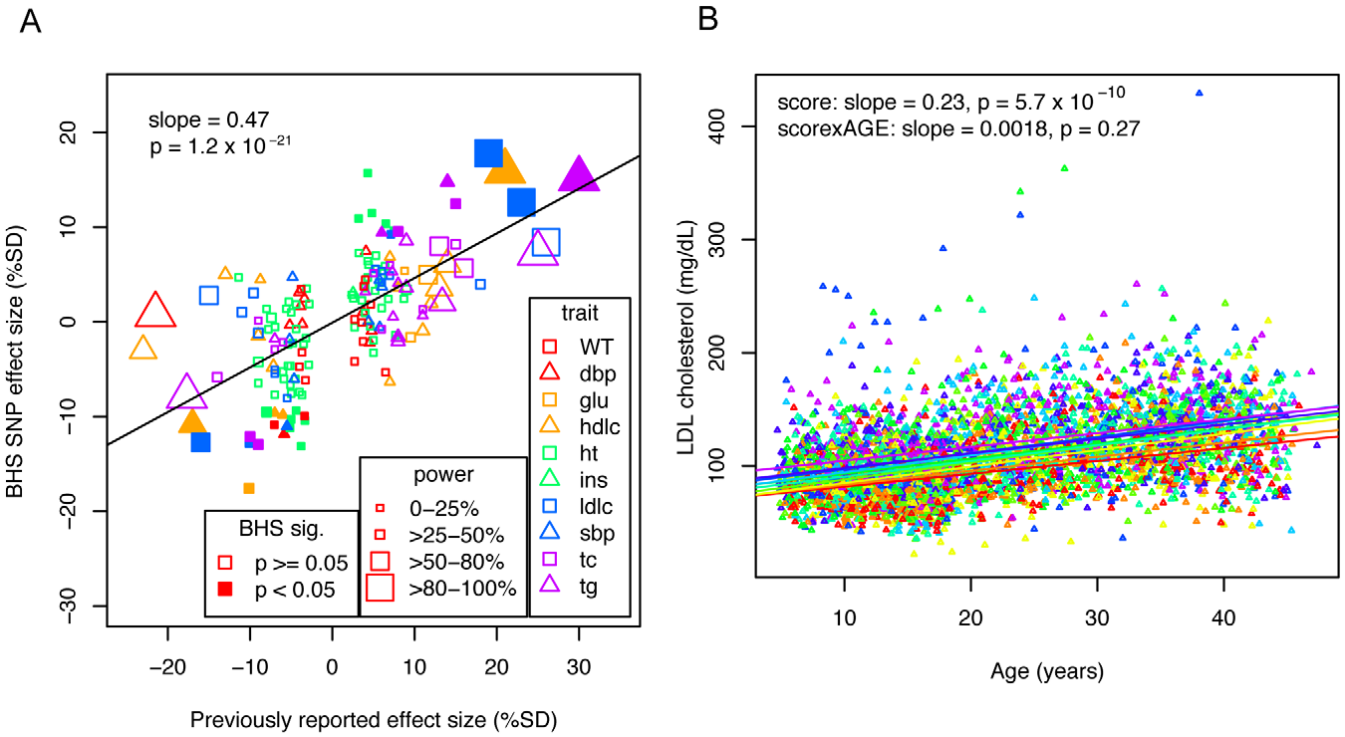
Effects of previously identified variants in the BHS. A) Effect sizes of previously identified markers are plotted against observed effects in the BHS. SNP-trait associations are plotted by shape and color to indicate trait. The size of the point indicates the power to detect an association of the magnitude previously described, and whether a point is filled in or not indicates whether the association was significant at *P*<0.05 in the BHS. Linear regression lines are shown, with the slope and p-value of the association between previously reported effect sizes and the observed effect sizes. B) Individuals were scored based on the effect size of each previously identified marker and are grouped and color-coded based on the decile of their score. Linear lines are linear regression estimates of the average trajectory of each decile group.

We assessed whether associations that have been described in previous adult cross-sectional GWA studies exhibit consistent effects over time and whether the effect sizes observed in children through middle-aged adults are consistent with those previously described. We identified 169 SNP-trait associations (see Materials and Methods) for which we had directly genotyped or imputed genotype data. We first estimated our power to detect each previous association at alpha = 0.05 under a more structured, but similar study design (i.e., 8 equally spaced measurements), given the previously reported effect size and allele frequency. Under this model, we would expect to have detected 40/169 (24%) associations at P<0.05, and we observed a similar number of SNP effects in the BHS data (32/169; 19%). We evaluated the associations across all traits together by comparing how well the previously reported effect size was recapitulated in the BHS GWA (Figure 1A). For consistency across studies and traits, if an effect size was not already expressed in terms of percent standard deviation (%SD), we converted the previously reported effect size into %SD and compared the previous effect size to the SNP effect. The previously reported effect size was a strong predictor of the SNP effect (slope = 0.47, *P* = 1.2×10^−21^), suggesting that SNPs that have been previously identified in adult cross-sectional GWA studies are good predictors of time-averaged effects in the BHS sample.

We also determined whether the same previously identified SNPs were likely to show effects on a trait over time (SNPxAGE effects). Under a simple model that assumed that all of the effect in adults is due to a locus that has no effect in childhood, we estimated power to detect such an interaction effect in a similarly structured study with 8 repeated measures. Given these assumptions, we would have expected to see 24/169 (14%) SNPxAGE associations. We observed 6/169 (3.6%) SNPs that showed SNPxAGE effects at P<0.05, indicating that effects seen in SNPs described in adult GWA studies are not due primarily to differences in effects over time, although larger studies will be required to definitively characterize this.

### Composite scoring

We considered whether a composite genotype score would better predict overall CVD risk factor trajectories or time-dependent effects than any single locus. For each person and each trait, we created a score by summing the expected effect in percent standard deviation of each allele that the person carried. We then determined whether the score was associated with the trait's average value and trajectory by using this score as a predictor for each trait in a linear mixed model, adjusting for age and gender. We assessed the score's average effect across time (score effect) and whether or not there was a time-dependent effect (score*age effect). The traits HDL, LDL, total cholesterol, triglycerides, and height showed strongly significant score effects, while only triglycerides showed a score*age effect (Table 5). Longitudinal data was visualized by color-coding the individuals according to the decile of their overall score and the average linear trend of each group was plotted (LDL, Figure 1B and others in Figure S3). These results indicate that the cumulative effects of SNPs that are identified in large adult cross-sectional studies are generally age-independent effects, with an exception in triglycerides, which was the only trait to show a significant score*age effect. We additionally tested whether previously identified variants were predictive of adult levels after adjusting for childhood levels (Table 6). We saw that 25/169 (14.8%) showed association at *P*<0.05. These observations in the BHS data suggest that even though results from existing GWA studies demonstrate age-independent effects, they can be predictive of trait values in adults.

**Table 5:** *P*-values associated with score and score*age effects (Age and Sex adjusted).

	Score	Score×AGE
**weight**	0.03	0.11
**diastolic BP**	0.62	0.92
**HDL-cholesterol**	1.9×10^−5^	0.11
**height**	8.8×10^−12^	0.86
**LDL-cholesterol**	5.7×10^−10^	0.27
**systolic BP**	0.11	0.21
**total cholesterol**	6.6×10^−9^	0.21
**triglycerides**	4.1×10^−8^	2.9×10^−5^

Scores based on the genotypes of previously reported SNPs were used to test for association with effects across time (Score) or time-dependent effects (Score×Age).

**Table 6:** *P*-values for predictive ability of previously identified SNPs, after adjusting for first measure in childhood.

Trait	SNP	Alleles	BETA	SE	*P*
diastolic BP	rs653178	T/C	−1.2	0.5	0.025
glucose	rs560887	T/C	−3.0	1.5	0.046
HDL-cholesterol	rs1532624	A/C	2.1	0.7	0.005
HDL-cholesterol	rs964184	G/C	−2.8	1.1	0.015
HDL-cholesterol	rs157580	A/G	−1.6	0.7	0.027
HDL-cholesterol	rs7395662	A/G	−1.5	0.7	0.038
HDL-cholesterol	rs471364	T/C	2.2	1.1	0.049
height	rs185819	T/C	0.9	0.3	0.003
height	rs3748069	A/G	0.9	0.3	0.006
height	rs710841	C/T	−0.8	0.3	0.013
height	rs4896582	A/G	−0.8	0.3	0.013
height	rs967417	G/A	0.7	0.3	0.013
height	rs757608	G/A	−0.7	0.3	0.031
height	rs3760318	A/G	−0.6	0.3	0.032
height	rs16896068	A/G	−0.8	0.4	0.037
height	rs6060373	A/G	−0.6	0.3	0.048
LDL-cholesterol	rs12740374	G/T	6.3	2.4	0.009
LDL-cholesterol	rs12272004	A/C	−8.7	4.1	0.034
systolic BP	rs3184504	C/T	−1.6	0.7	0.023
total cholesterol	rs693	G/A	−5.7	2.3	0.013
total cholesterol	rs2304130	A/G	8.9	4.1	0.031
triglycerides	rs964184	G/C	51.7	10.7	1.9×10^−6^
triglycerides	rs780094	C/T	−19.1	7.2	0.009
triglycerides	rs7819412	A/G	13.4	6.7	0.045
weight	rs7138803	G/A	−2.2	1.0	0.037

Previously associated SNPs were tested for whether they were associated with adult level traits after adjusting for the trait level seen in childhood. Only associations at *P*<0.05 are shown.

## Discussion

We identified seven associations at *P*<5×10^−8^ showing either time-averaged or time-dependent effects on CVD risk factors in the BHS, two of which have been previously characterized. Of all associations with *P*<10^−6^, we were able to strongly replicate the association in the YF with HDL-cholesterol at *CETP* with a combined *P* = 9.7×10^−24^, and LDL-cholesterol at *APOE* with a combined *P* = 8.7×10^−19^. Differences that exist between the cohorts, such as birth year (15 year difference), and environmental differences could have influenced replication of the remaining SNPs. Larger discovery studies will also have better resolution and power to accurately estimate longitudinal effect sizes, likely allowing for more robust replication.

We evaluated the longitudinal effects of markers that have been previously identified in adult GWA studies. We found that previously identified markers showed time-averaged effects consistent with their reported effect size. This argues that the linear mixed model is an effective tool for modeling time-averaged effects in a GWA setting and that adult GWA studies may be capturing variation that tends to have consistent effects over time. Using a scoring approach, the overall signal from previously identified markers tended to have strong associations with time-averaged effects, but except in the case of triglycerides, did not show time-dependent effects. Previously identified markers were also likely to be associated with adult trait levels above and beyond childhood levels. Although we primarily describe time-averaged effects for previously identified markers, there may be more subtle time-dependent effects that larger studies will be better able to capture.

It is important to note that although we focused on analysis of linear trends over time, a linear model may not best capture these trends. Other approaches could be explored further such as non-linear models when there is an *a priori* expectation of trait trajectory, or model free approaches. These additional models could lead to additional variations that influence trajectories, or more precise estimations of effect size.

Longitudinal studies are particularly suited to capturing effects that vary over time. Genetic variation that shows a time-dependent effect may help predict those that will go onto develop disease before they show symptomatic traits. The discovery of variants associated with SNPxAGE interaction effects could thus be used to screen young individuals who are pre-symptomatic and provide the opportunity for preventive measures decades before disease onset. We explored how well the markers that we identified predicted adult traits after correcting for childhood traits and suggest further study of variants with SNPxAGE effects as possibly better predictors of adult trait levels above and beyond childhood levels. These results are consistent with the idea that longitudinal studies may be a useful tool to better capture time-dependent variation that could ultimately be better predictive of future outcomes.

## Materials and Methods

### Ethics statement

The study was approved by the institutional review board and the ethics committee of each institution. Written informed consent was obtained from each participant in accordance with institutional requirements and the Declaration of Helsinki Principles. All subjects in the BHS gave informed consent at each examination, and for those under 18 years of age, consent of a parent/guardian was obtained. Study protocols were approved by the Institutional Review Board of the Tulane University Health Sciences Center.

### The Bogalusa Heart Study (BHS)

Between 1973 and 2008, 9 cross-sectional surveys of children aged 4–17 years and 10 cross-sectional surveys of adults aged 18–48 years (Figure S4), who had been previously examined as children, were conducted for CVD risk factor examinations in Bogalusa, Louisiana. This panel design of repeated cross-sectional examinations has resulted in serial observations from childhood to adulthood. By linking the 19 surveys, 12,163 individuals have been examined, with 37,317 observations. In the ongoing Longitudinal Aging Study funded by NIH and NIA since 2000, there are 1,202 subjects who have been examined 4–14 times from childhood to adulthood and have DNA available for GWA genotyping. Based on the analysis of identity-by-state (IBS) sharing from whole genome genotyping data, we focus on a subset of 525 genotyped individuals who are of European ancestry and unrelated (229 male, 296 female). The average number of measurements per individual is 8 (range 4–13).

### The Cardiovascular Risk in Young Finns Study (YF)

The YF cohort is a Finnish longitudinal population study sample on the evolution of cardiovascular risk factors from childhood to adulthood [21]. The first cross-sectional study was conducted in 1980 in five centers and included 3,596 participants in the age groups of 3, 6, 9, 12, 15, and 18, who were randomly chosen from the national population register. After baseline in 1980 these subjects have been re-examined in 1983 and 1986 as young individuals, and in 2001 and 2007 as older individuals. Genotype data for the present analysis (DNA collected in 1980, 2001 and 2007) was available for 2,442 individuals.

In the latest follow-up in 2001, a total of 2,283 participants (of which DNA is available from 2,265 participants) were examined for numerous study variables, including serum lipoproteins, glucose, insulin, obesity indices, blood pressure, life-style factors, smoking status, alcohol use and general health status.

### Genotyping & QC

#### BHS genotyping

We genotyped 1,202 BHS samples using the Illumina Human610 Genotyping BeadChip [22], and HumanCVD BeadChip [11]. Genotypes were called using a clustering algorithm in Illumina's BeadStudio software. Three samples on the Human610 BeadChip gave poor results (call rates <99%) and were discarded from the study. In addition, 3 samples had a different estimated gender from genotype data versus gender provided with the phenotype data and were also discarded. SNPs with call rates <90% were discarded, and SNPs with call rates between 90–95% or cluster separation score <0.3 were manually inspected and cluster positions were edited if needed. We removed approximately 30,000 SNP loci (4.9%) due to poor performance. The final average sample call rate was 99.95% for the Human610 BeadChip, and 99.32% for the CVD BeadChip. We assessed reproducibility by genotyping 29 samples in duplicate (18 known replicates, 11 blind replicates), and observed >99.99% identical genotype calls on both BeadChips. Finally we observed 99.98% genotype concordance in 12,581 overlapping SNPs between the 610 and CVD BeadChips. A final list of 545,821 unique SNPs passed QC and allele frequency filters.

#### YF study genotyping

For replication, we had genome-wide SNP data from a custom Illumina BeadChip containing 670,000 SNPs and CNV probes from 2,442 YF participants (1,123 males, 1,319 females). The custom content on the custom 670K array replaced some poor performing SNPs on the Human610 BeadChip and added more CNV content, and includes 546,677 SNPs passing QC from 594,210 SNPs on the chip. The custom 670K chip shares 562,643 SNPs in common with the Illumina Human610 BeadChip. Genotypes were called using Illumina's clustering algorithm [23]. A total of 2,556 samples were genotyped. After initial clustering, we removed 2 subjects for poor call rates (CR<0.90), and 54 samples failed subsequent QC (i.e., duplicated samples, heterozygosity, low call rate, or custom SNP fingerprint genotype discrepancy). The following filters were applied to the remaining data: MAF 0.01, GENO 0.05, MIND 0.05, and HWE 1×10^−6^. Three of 2,500 individuals were removed for low genotyping (MIND>0.05), 11,766 markers were excluded based on HWE test (*P*≤1×10^−6^), 7,746 SNPs failed missingness test (GENO>0.05), 34,596 SNPs failed frequency test (MAF<0.01) and one individual failed gender check. A final list of 546,677 SNPs passed QC and allele frequency filters.

#### Assessing cryptic relatedness

Bogalusa participants with genotype data were filtered for relatedness. Whole-genome genotype data was used to calculate identity-by-descent (PI_HAT) values in PLINK [24]. Individuals were then removed such that no pair of individuals retained a PI_HAT value greater than 0.10. PI_HAT values were consistent with known sibling and half-sibling relationships. The final list consisted of 525 BHS individuals.

In the YF data, there were 546,770 SNPs and 2,496 individuals which were utilized to generate an identity-by-descent (IBD) matrix file in PLINK [24]. There were 51 pairs of individuals with pi-hat greater than 0.2 thus these individuals removed due to possible relatedness. One of the pair was removed using greater missingness as criteria. The final list consisted of 2,442 YF subjects.

#### Imputation

We imputed genotypes in genotyped BHS individuals for all HapMap (phase II, release 22) SNPs using the program MACH [12]. The best estimate of the quantitative allele dosage was used as the predictor in association tests. The CEU HapMap phased haplotypes were used as a reference (N = 60 unrelated individuals). This resulted in overall allelic error rates of 1.6%. SNPs were filtered for minor allele frequency (<5%) and r^2^ with respect to genotyped SNPs (<0.30), resulting in genotype data in a total of 2,173,391 SNPs. Imputation was performed in the YF samples using MACH with the HapMap release 22 CEU haplotypes as reference.

### Prediction ability

Previously identified markers were obtained through the NHGRI database [25] (accessed 5/20/09). Marker associations, alleles, and allele frequencies were verified with those reported in the original papers and corrected if required. Markers were used if the alleles at the locus provided unambiguous orientation or if the allele frequencies were different enough between A/T and C/G SNPs to distinguish which allele was the associated allele. We thus excluded any A/T or C/G SNPs with a minor allele frequency >0.4 and required that the allele frequency in the previously reported study be within 10% of the allele frequency in the BHS. We excluded studies of non-European Ancestry origin. One SNP per cytogenic region was used for each phenotype: the SNP with the smallest previously reported p-value was used.

Effect size was translated to percent standard deviation. If the effect size was reported in an absolute measure (e.g. cm for height), then the standard deviation from the BHS study was used. Standard deviation was calculated from the standard error of the SNP association reported in the linear mixed model. For glucose, cholesterol, and triglycerides measures, units were converted to mg/dl before converting to %SD.

A risk value was calculated for each individual based on the imputed genotype and previously reported effect size, converted to %SD. The %SD was multiplied by the allelic dosage for each SNP and summed over all the associated SNPs for each phenotype. The resulting risk value was then used as a predictor for the BHS individuals.

### Genome-wide association

GWA was performed using linear mixed model regression with fixed covariates of age and sex, random slope, and random intercept. Genotypes were coded as 0,1, or 2 when the SNP was genotyped and by dosage (scale 0–2) when imputed. Analysis was performed within the nlme package in R [13]. Covariance structures were determined by testing all spatial covariance structures (exponential, Gaussian, linear, rational quadradics, and spherical) with covariates and a sample of SNPs, and picking the structure that best fit the data as measured by the lowest AIC (Akaike Information Criteria) value. SNP and SNPxAGE interaction effects were estimated separately. Although the default nlme optimizer tended to have difficulty converging, we obtained good results by using the optim optimizer on data where all missing data was removed. The number of SNPs that converged and for which we obtained results is listed in Table S1. Analyses were performed on a compute cluster with 600,000 tests taking ∼3 hrs on 64 processors.

### Filtering for genomic inflation

If genomic inflation factors were inflated or deflated, we reran the GWA using the first four MDS components as covariates. If the inflation factor was still less than 0.90 or greater than 1.05, we removed the analysis. In addition, we filtered body mass index (BMI) SNP, BMI SNPxAGE, and weight SNP analyses completely from the analysis due to a combination of consistently inflated or deflated genomic inflation factors or a long list of highly associated SNPs.

### Power

Power was calculated using G*Power 3 [26].We used the MANOVA repeated measures module with 8 repeated measures with a correlation of 0.5 between them, similar to the correlations seen in this study. We estimated power for between-factor and between-within interaction effects. Effect size (f) was calculated as
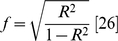
and R^2^ was calculated from the allele frequencies as reported in the original associations (p and q) and the effect size in terms of %SD [27].

## Supporting Information

Figure S1 Regional plots of top SNP and SNPxAGE associations. Regions are ordered by phenotype and significance as in Table 1 and 2. SNPs are indicated by triangle (directly genotyped) or square (imputed), and colored according to LD (r^2^) with the top SNP with increasing shades of red indicating stronger LD. Blue lines indicate recombination hotspots and refSeq genes are indicated.
(8.26 MB PDF)

Figure S2 Manhattan Plots of GWAS results for each trait. Manhattan plots are shown for each SNP and SNPxAGE GWAS. Each point corresponds to an association with triangles indicating directly genotyped data and circles indicating imputed data. A horizontal line is plotted at *P* = 10^−6^ and SNPs above this point are outlined in pink. These SNPs occur in Tables 1, 2, and 3. Chromosomes are plotted in alternating blue and grey. *P*-values greater than 0.001 are not plotted.
(8.19 MB PDF)

Figure S3 Longitudinal profiles of cumulative score from previously identified SNPs. Individuals were scored based on the effect size of each previously identified marker as in Figure 1B. Individuals are grouped and color-coded based on the decile of their score. Linear lines were calculated using linear regression with all points from all individuals in a given decile.
(1.26 MB PDF)

Figure S4 Age at measurement in the BHS. All exam dates that were included in the study are plotted as a function of the age of participant at the exam date. Individuals had between 4–13 measurements. A single individual is highlighted in red.
(0.27 MB PDF)

Table S1 Number of SNPs that successfully converged and produced association statistics.
(0.06 MB XLSX)
