# Supplementary material for: Longitudinal Genome-Wide Association of Cardiovascular Disease Risk Factors in the Bogalusa Heart Study
Source: PLoS Genet. 2010 Sep 9;6(9):e1001094. doi: 10.1371/journal.pgen.1001094 (PMC2936521; doi:10.1371/journal.pgen.1001094)
Supplement: Figure S1 — Regional plots of top SNP and SNPxAGE associations. Regions are ordered by phenotype and significance as in Table 1 and 2. SNPs are indicated by triangle (directly genotyped) or square (imputed), and colored according to LD (r2) with the top SNP with increasing shades of red indicating stronger LD. Blue lines indicate recombination hotspots and refSeq genes are indicated. (8.26 MB PDF) [file pgen.1001094.s001.pdf]

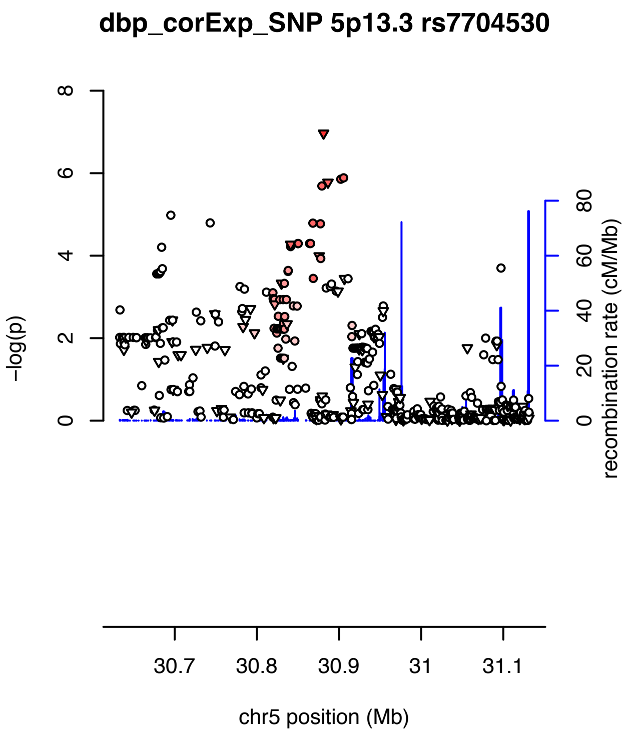
dbp_corExp_SNP 5p13.3 rs7704530
-log(p)
recombination rate (cM/Mb)
chr5 position (Mb)
30.7
30.8
30.9
31
31.1

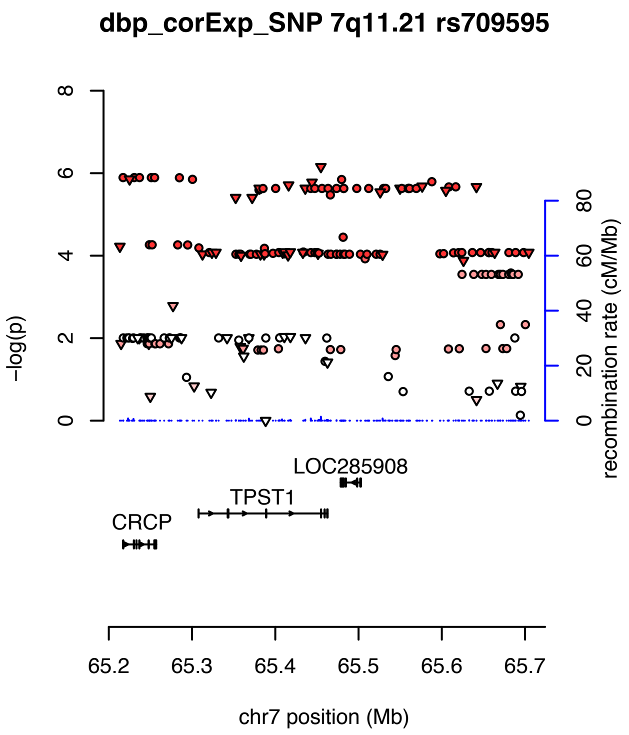
dbp_corExp_SNP 7q11.21 rs709595
-log(p)
recombination rate (cM/Mb)
LOC285908
TPST1
CRCP
chr7 position (Mb)
65.2
65.3
65.4
65.5
65.6
65.7

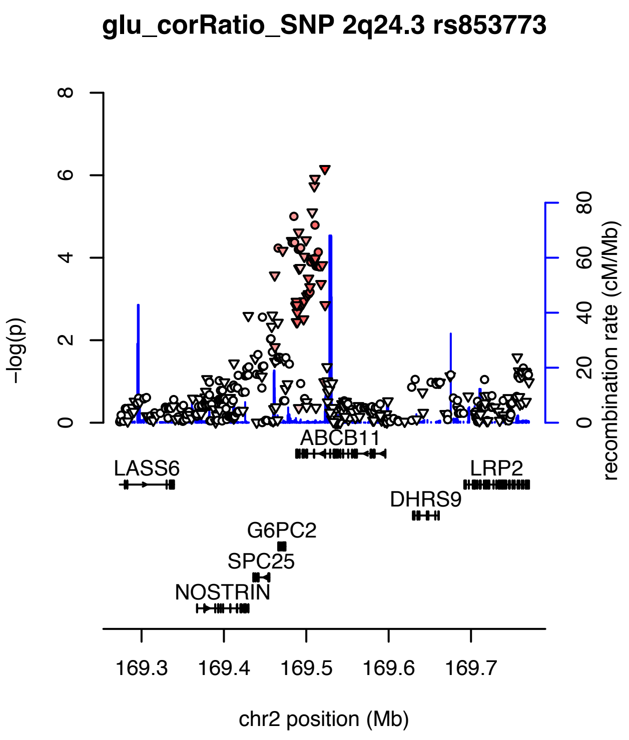
glu_corRatio_SNP 2q24.3 rs853773
-log(p)
recombination rate (cM/Mb)
ABCB11
LASS6
LRP2
DHRS9
G6PC2
SPC25
NOSTRIN
chr2 position (Mb)
169.3
169.4
169.5
169.6
169.7

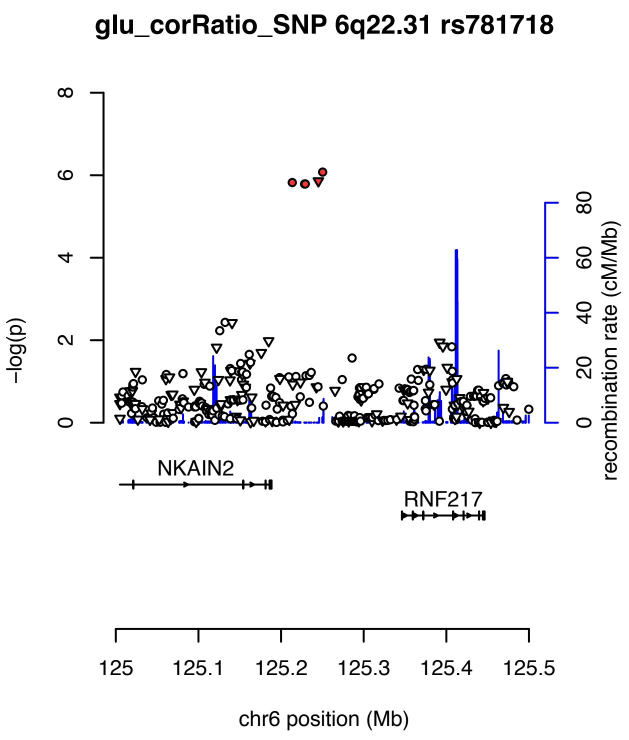
glu_corRatio_SNP 6q22.31 rs781718
-log(p)
recombination rate (cM/Mb)
NKAIN2
RNF217
chr6 position (Mb)
125
125.1
125.2
125.3
125.4
125.5

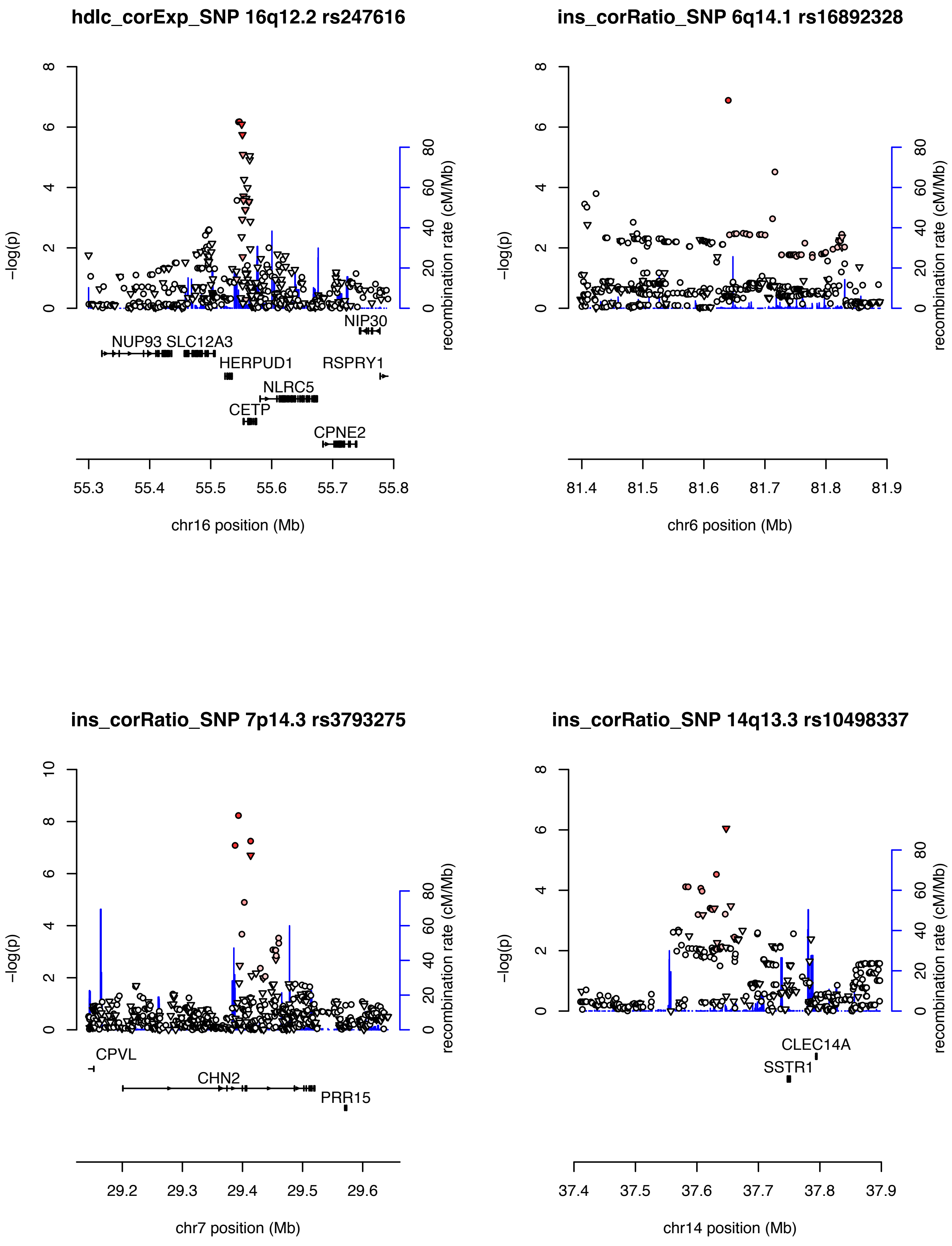

hdlc_corExp_SNP 16q12.2 rs247616
−log(p)
recombination rate (cM/Mb)
NIP30
NUP93 SLC12A3
HERPUD1
RSPRY1
NLRC5
CETP
CPNE2
chr16 position (Mb)
ins_corRatio_SNP 6q14.1 rs16892328
−log(p)
recombination rate (cM/Mb)
chr6 position (Mb)
ins_corRatio_SNP 7p14.3 rs3793275
−log(p)
recombination rate (cM/Mb)
CPVL
CHN2
PRR15
chr7 position (Mb)
ins_corRatio_SNP 14q13.3 rs10498337
−log(p)
recombination rate (cM/Mb)
CLEC14A
SSTR1
chr14 position (Mb)

ins_corRatio_SNP 18p11.31 rs1613695

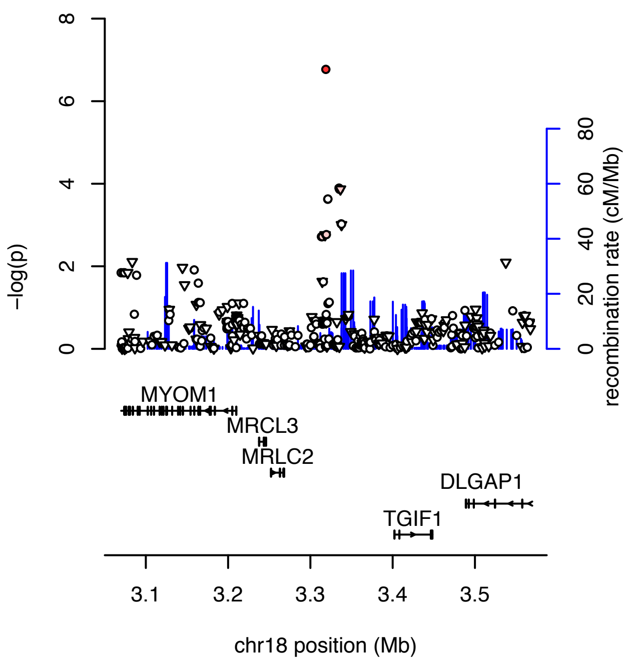

ins_corRatio_SNP 20p13 rs6052399

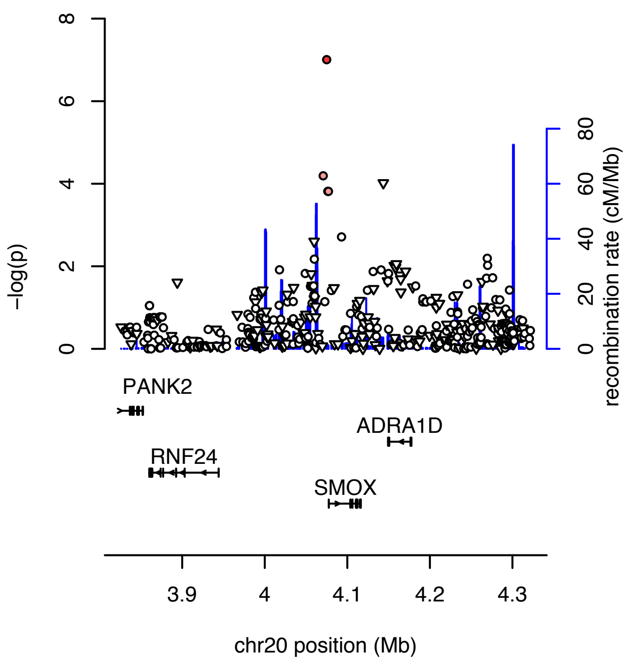

ldlc_corRatio_SNP 5q33.2 rs10044666

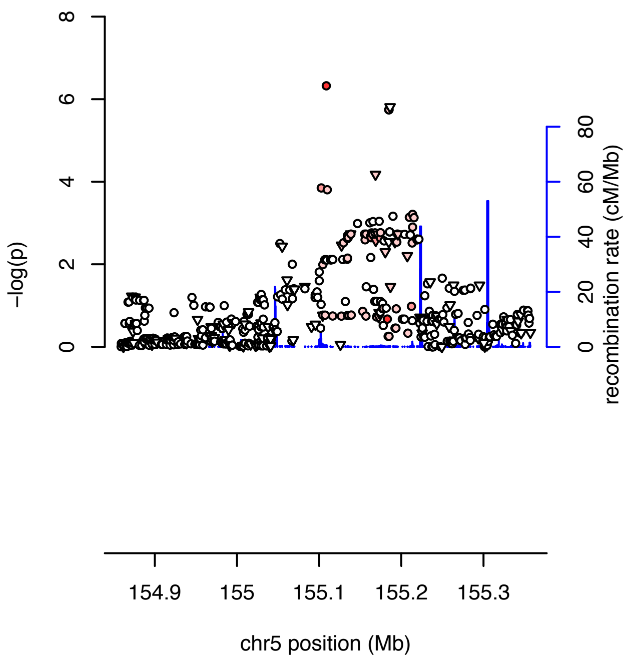

ldlc_corRatio_SNP 6q22.31 rs7738656

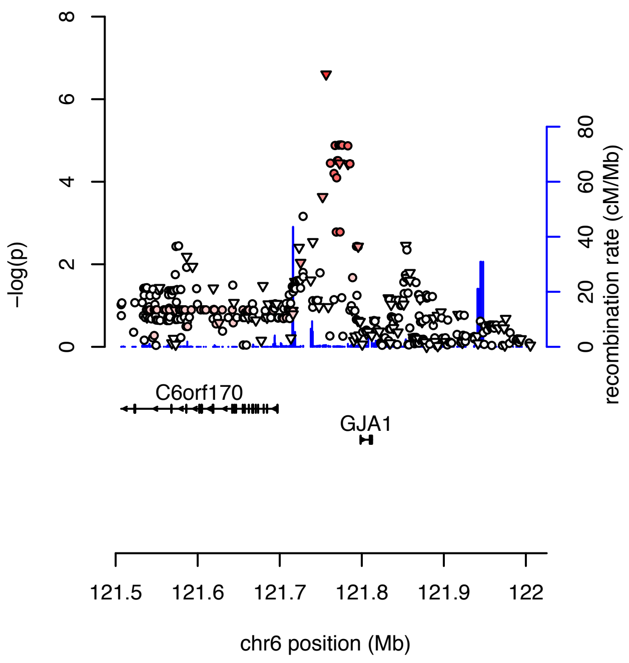

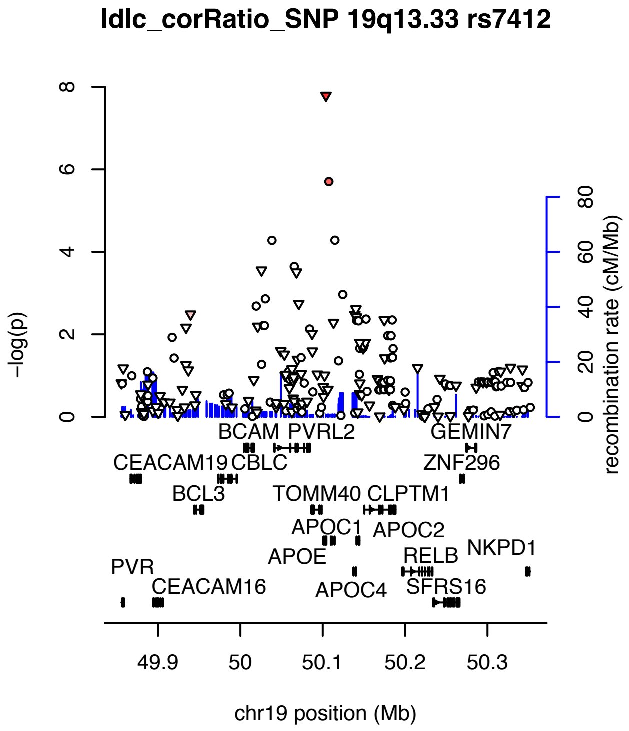
ldlc_corRatio_SNP 19q13.33 rs7412
−log(p)
recombination rate (cM/Mb)
BCAM PVRL2
GEMIN7
CEACAM19 CBLC
ZNF296
BCL3
TOMM40 CLPTM1
APOC1
APOC2
APOE
RELB
NKPD1
PVR
CEACAM16
APOC4
SFRS16
chr19 position (Mb)

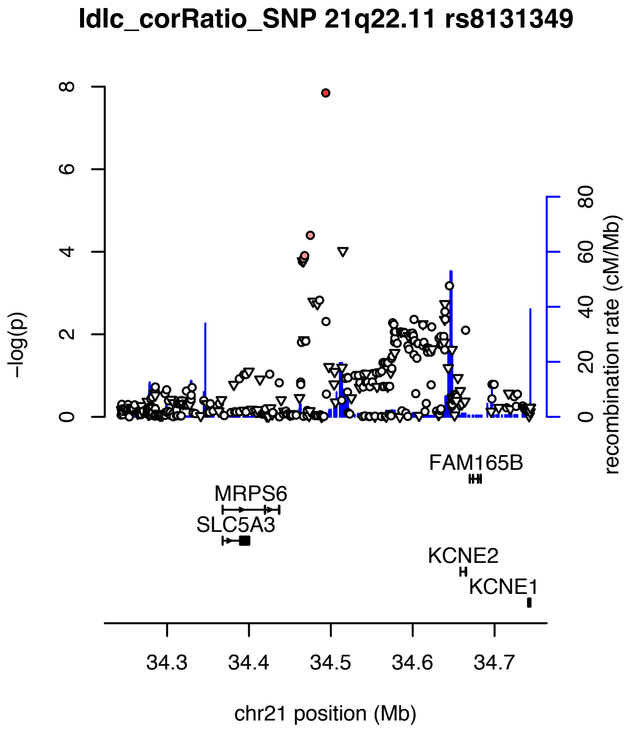
ldlc_corRatio_SNP 21q22.11 rs8131349
−log(p)
recombination rate (cM/Mb)
FAM165B
MRPS6
SLC5A3
KCNE2
KCNE1
chr21 position (Mb)

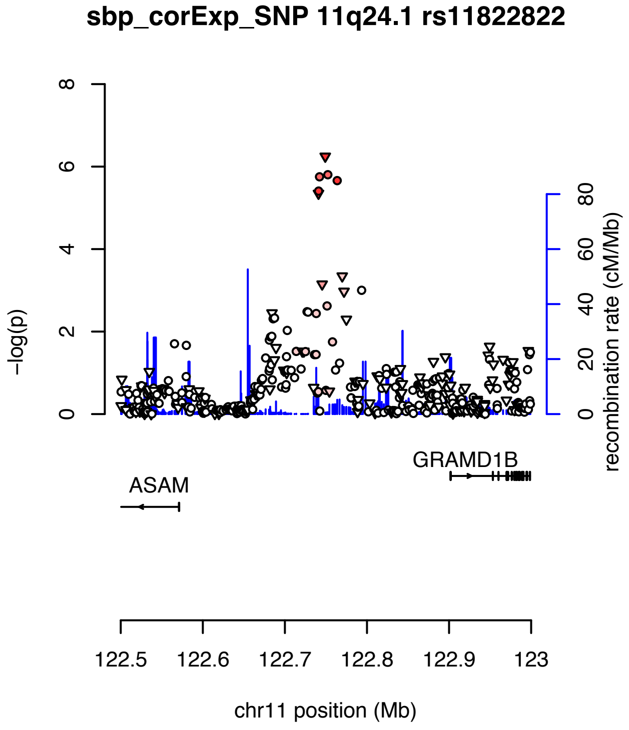
sbp_corExp_SNP 11q24.1 rs11822822
−log(p)
recombination rate (cM/Mb)
GRAMD1B
ASAM
chr11 position (Mb)

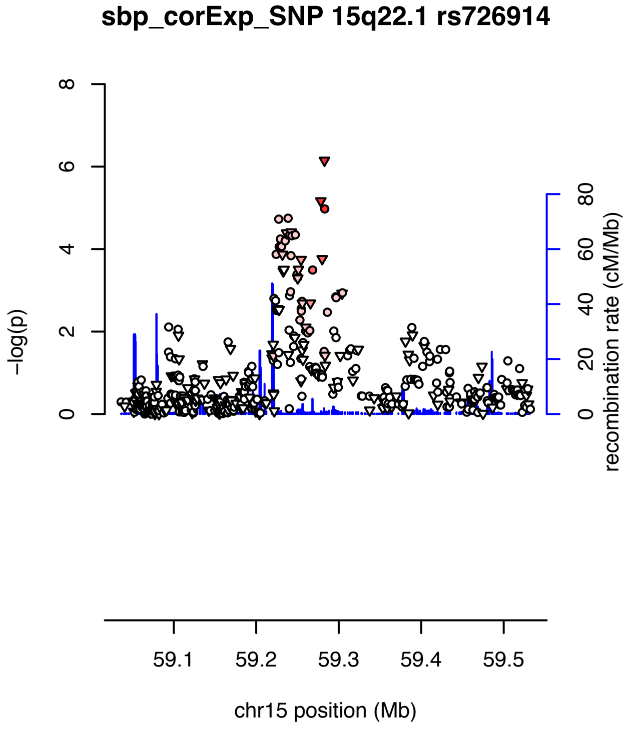
sbp_corExp_SNP 15q22.1 rs726914
−log(p)
recombination rate (cM/Mb)
chr15 position (Mb)

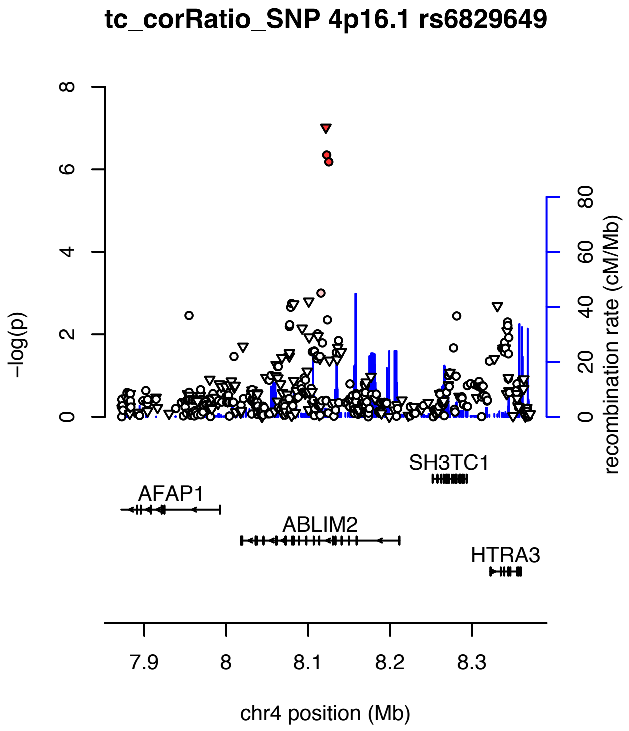

tc_corRatio_SNP 4p16.1 rs6829649
−log(p)
recombination rate (cM/Mb)
SH3TC1
AFAP1
ABLIM2
HTRA3
chr4 position (Mb)

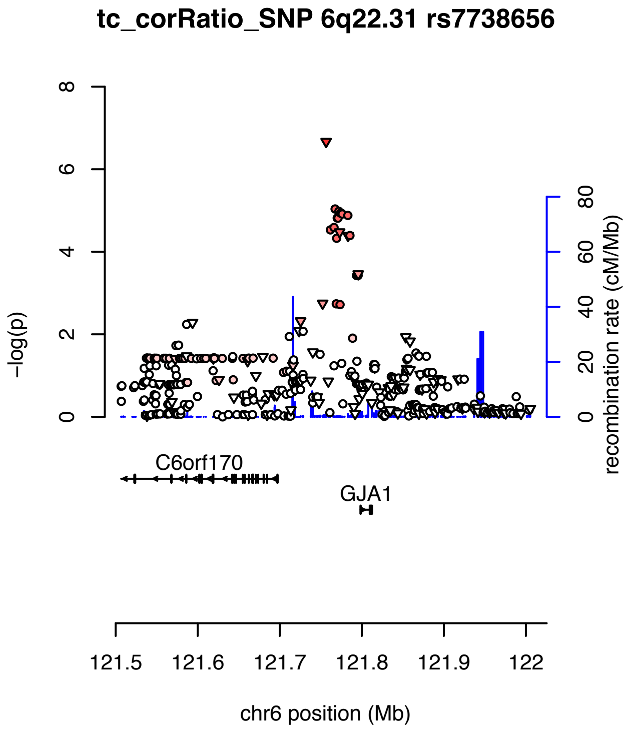

tc_corRatio_SNP 6q22.31 rs7738656
−log(p)
recombination rate (cM/Mb)
C6orf170
GJA1
chr6 position (Mb)

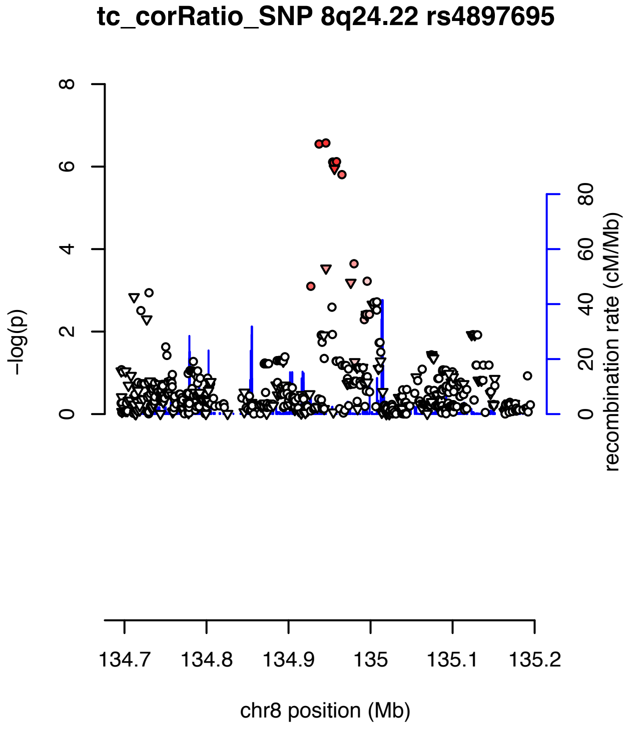

tc_corRatio_SNP 8q24.22 rs4897695
−log(p)
recombination rate (cM/Mb)
chr8 position (Mb)

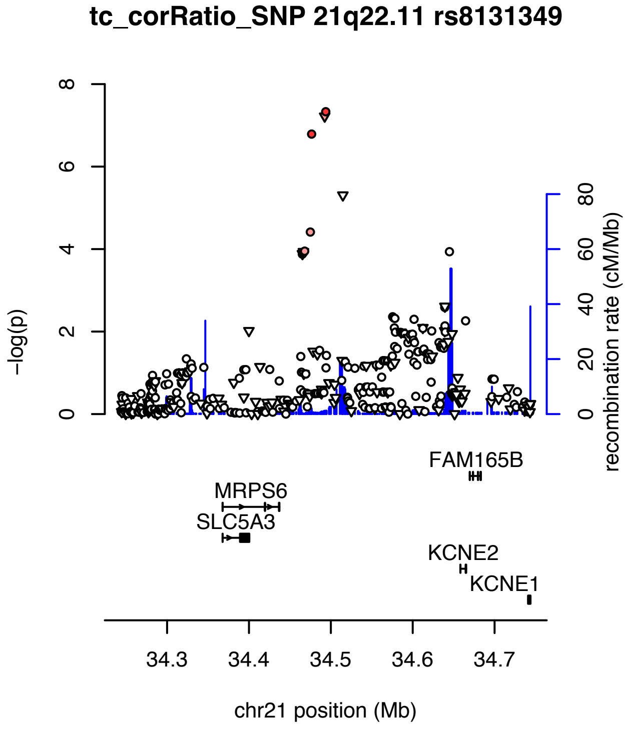

tc_corRatio_SNP 21q22.11 rs8131349
−log(p)
recombination rate (cM/Mb)
FAM165B
MRPS6
SLC5A3
KCNE2
KCNE1
chr21 position (Mb)

WAIST_corRatio_SNP 8q24.13 rs891541

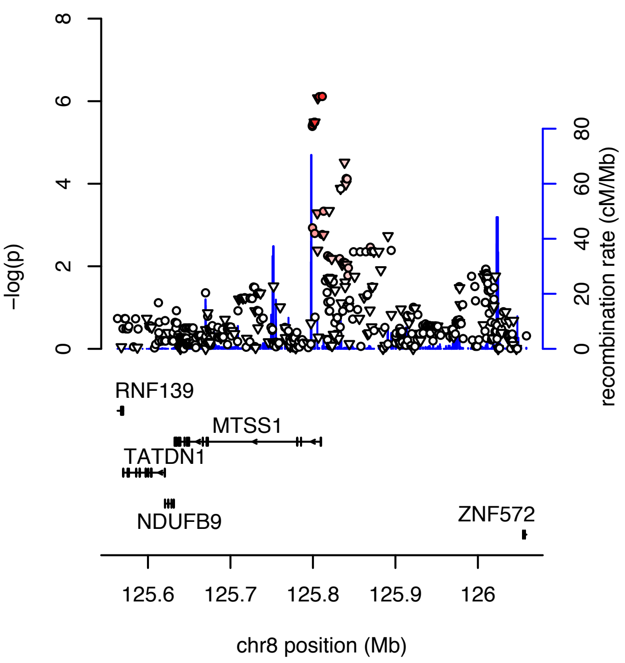

dbp_corExp_SNPxAGE 14q24.3 rs17104804

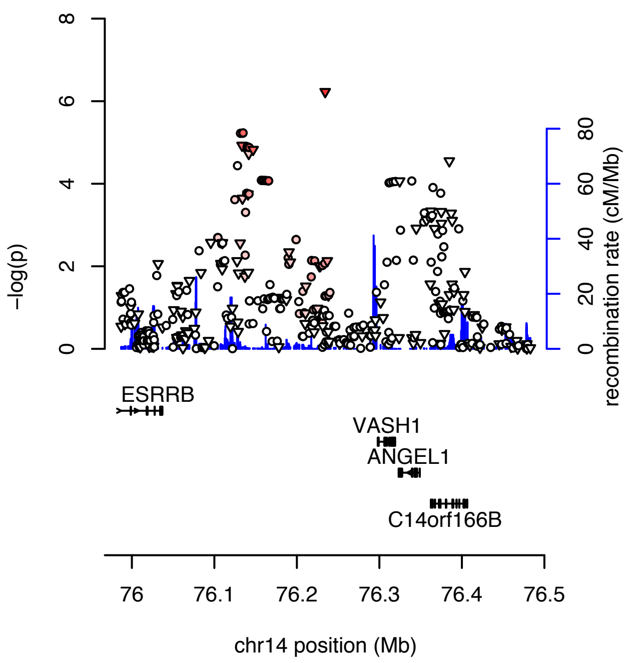

glu_corRatio_SNPxAGE 1q32.1 rs12069004

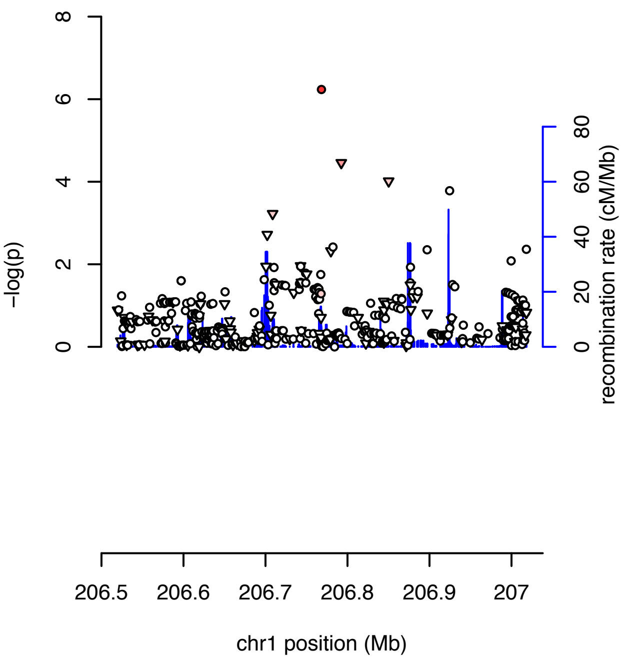

glu_corRatio_SNPxAGE 7p14.3 rs3793275

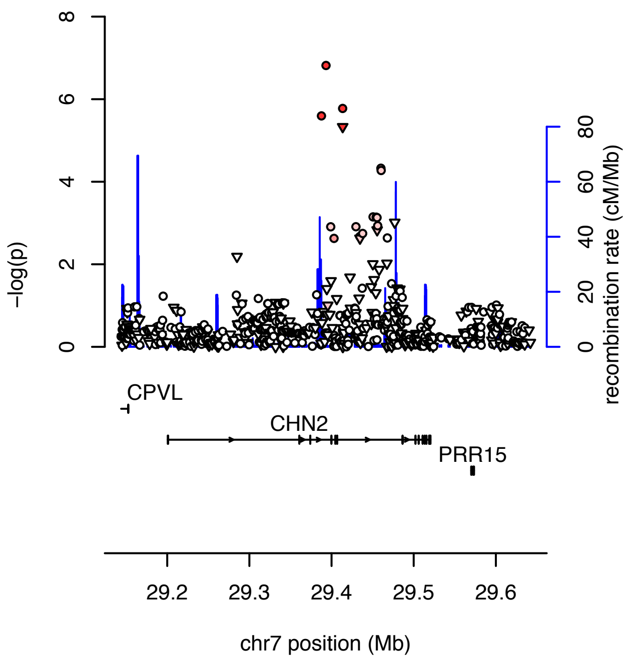

glu_corRatio_SNPxAGE 7p21.1 rs10260737

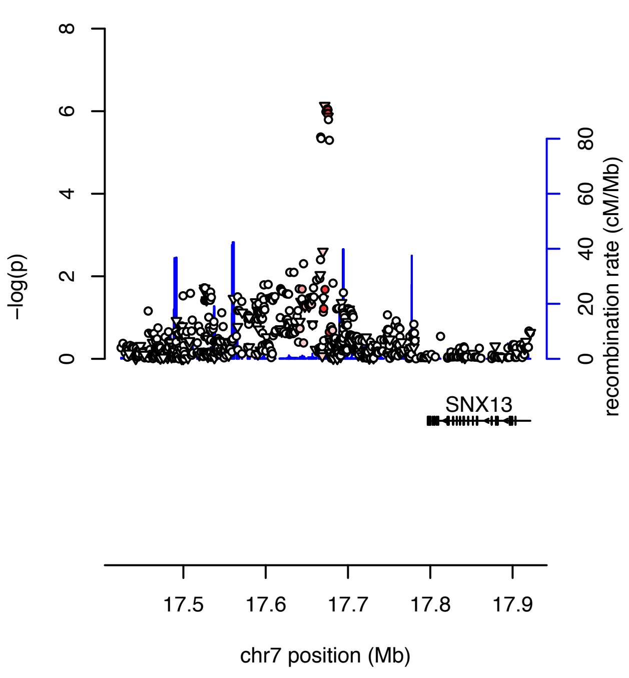

glu_corRatio_SNPxAGE 8q23.2 rs3019325

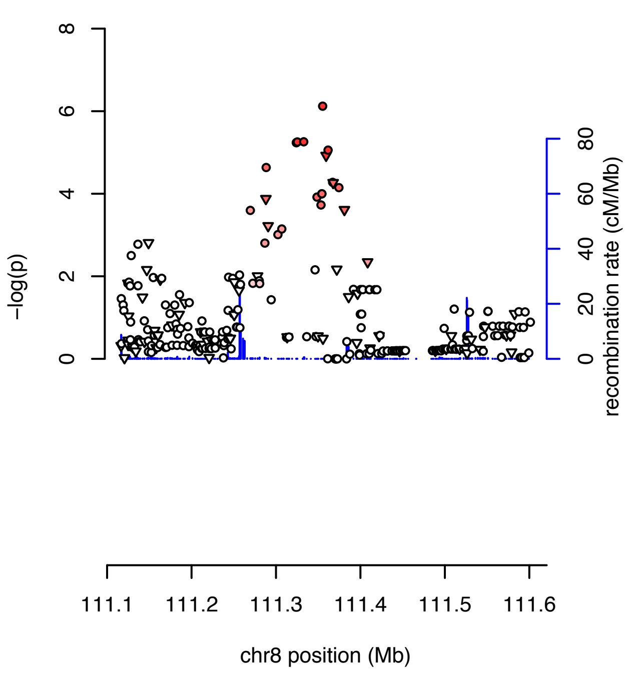

glu_corRatio_SNPxAGE 8q24.22 rs12548494

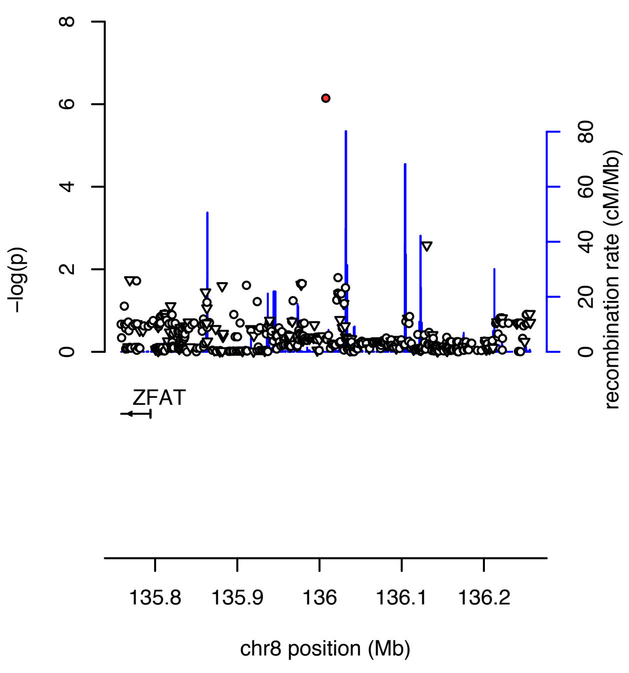

glu_corRatio_SNPxAGE 11q13.5 rs12807555

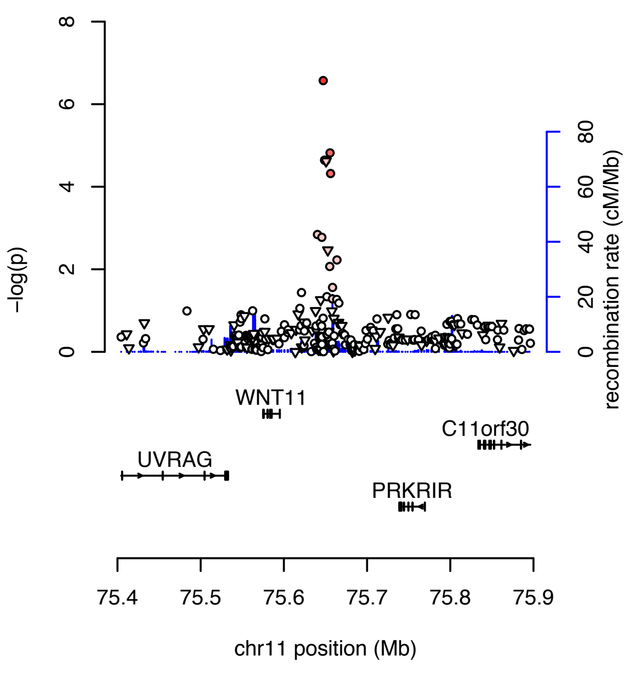

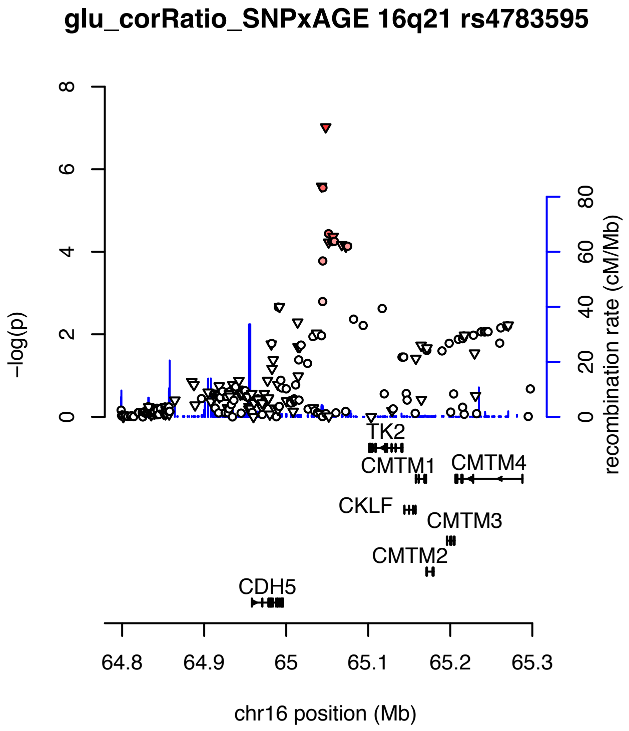
glu_corRatio_SNPxAGE 16q21 rs4783595
−log(p)
recombination rate (cM/Mb)
TK2
CMTM1
CMTM4
CKLF
CMTM3
CMTM2
CDH5
64.8 64.9 65 65.1 65.2 65.3
chr16 position (Mb)

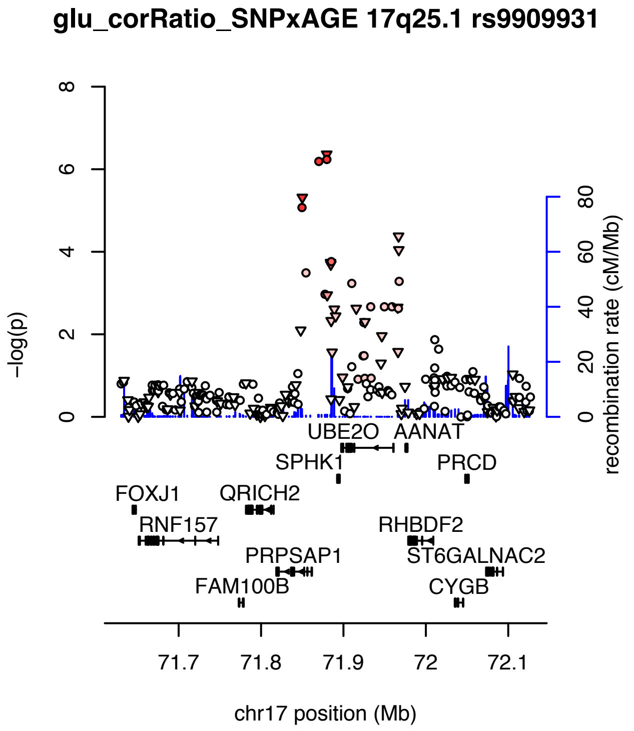
glu_corRatio_SNPxAGE 17q25.1 rs9909931
−log(p)
recombination rate (cM/Mb)
UBE2O
AANAT
SPHK1
PRCD
FOXJ1
QRICH2
RNF157
RHBDF2
PRPSAP1
ST6GALNAC2
FAM100B
CYGB
71.7 71.8 71.9 72 72.1
chr17 position (Mb)

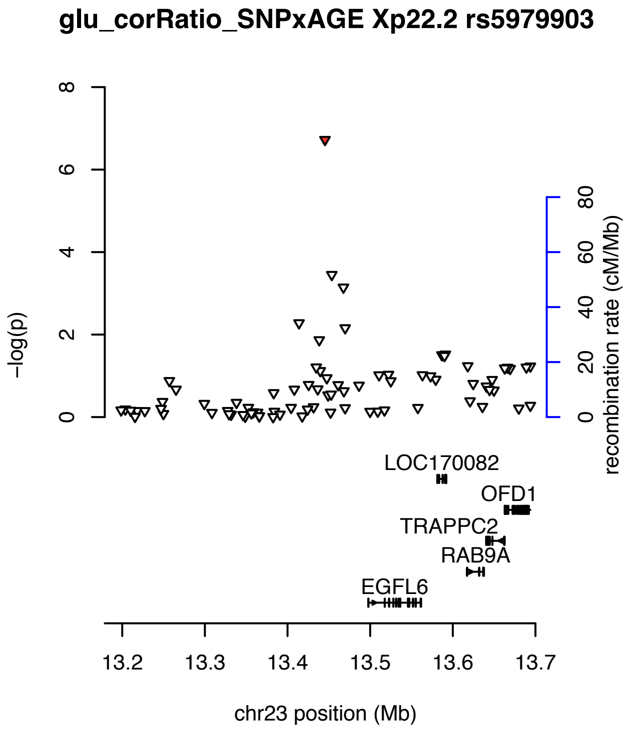
glu_corRatio_SNPxAGE Xp22.2 rs5979903
−log(p)
recombination rate (cM/Mb)
LOC170082
OFD1
TRAPPC2
RAB9A
EGFL6
13.2 13.3 13.4 13.5 13.6 13.7
chr23 position (Mb)

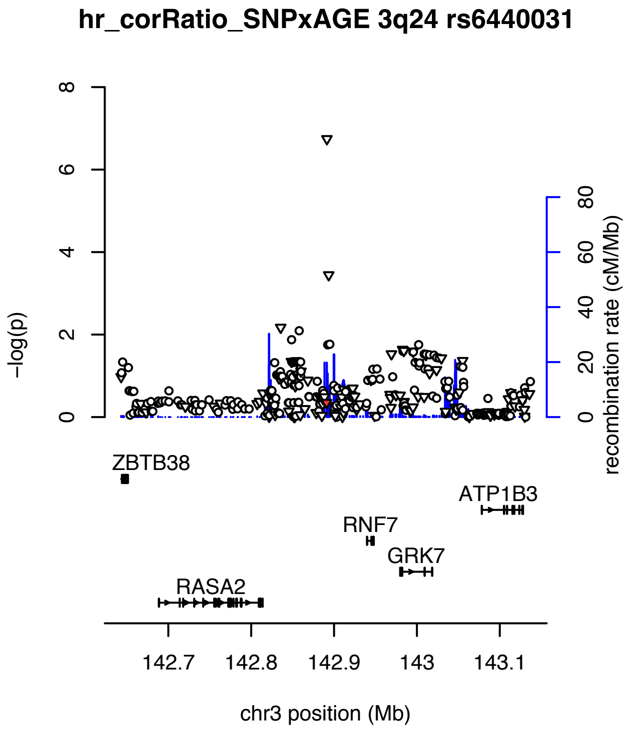
hr_corRatio_SNPxAGE 3q24 rs6440031
−log(p)
recombination rate (cM/Mb)
ZBTB38
ATP1B3
RNF7
GRK7
RASA2
142.7 142.8 142.9 143 143.1
chr3 position (Mb)

hr_corRatio_SNPxAGE 12q21.2 rs1449460

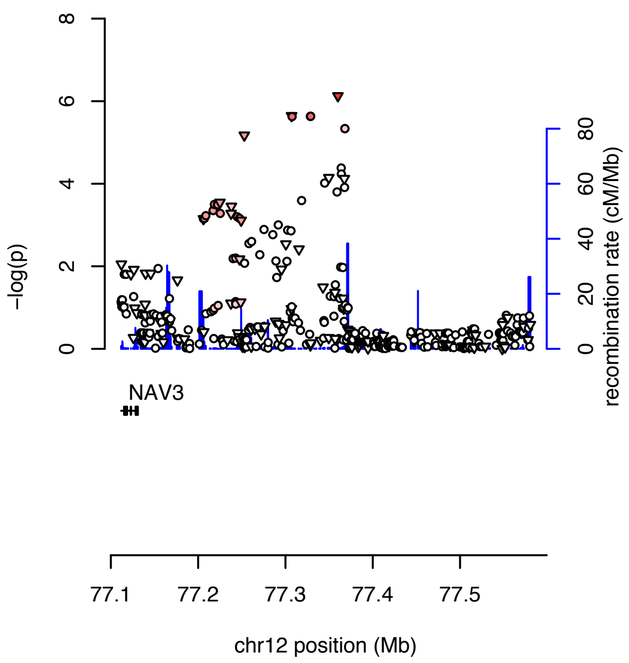

ht_corLin_SNPxAGE 11q23.3 rs1144036

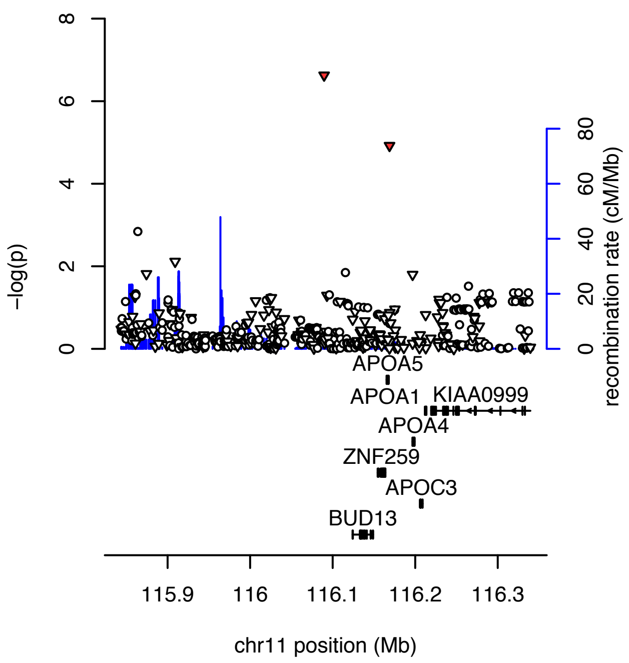

ht_corLin_SNPxAGE 19q13.32 rs8103264

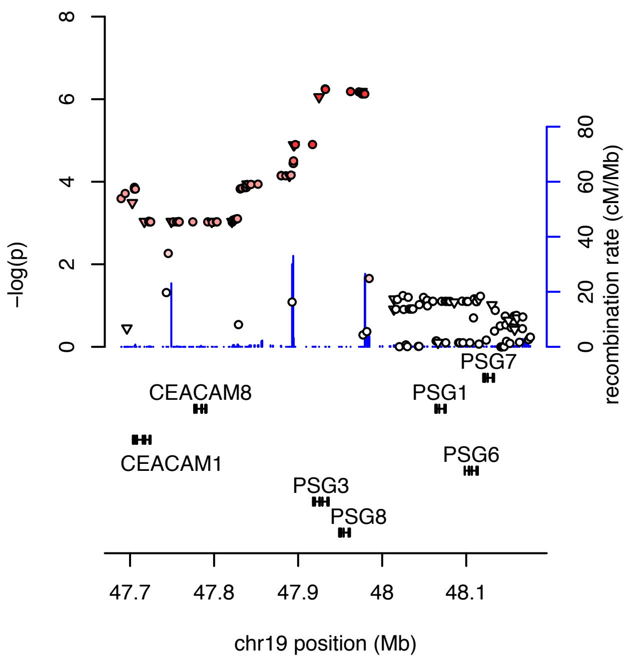

ins_corRatio_SNPxAGE 12p11.23 rs1552257

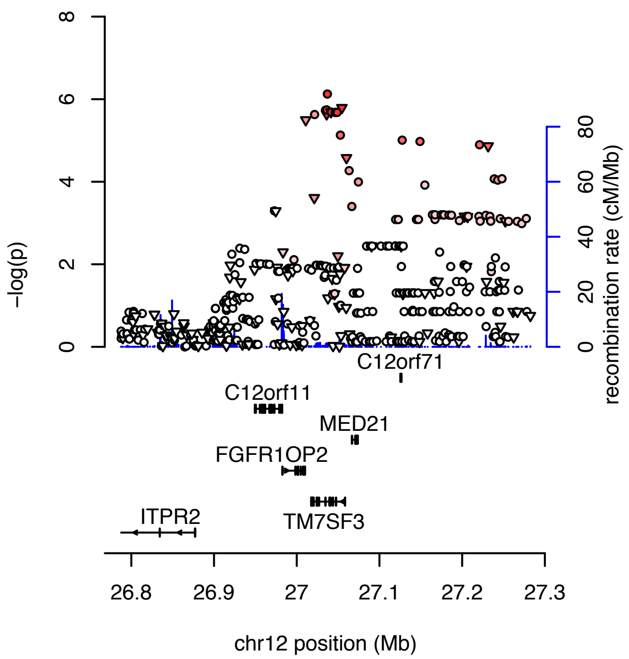

ldlc_corRatio_SNPxAGE 17q22 rs8073909

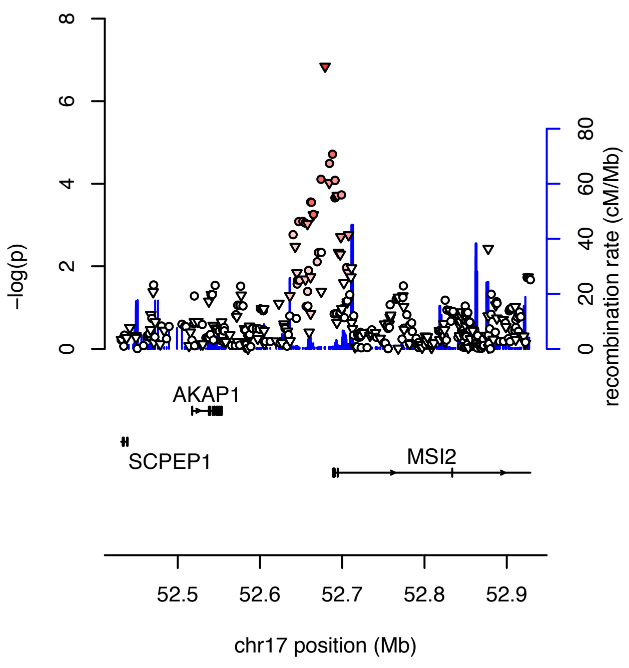

ldlc_corRatio_SNPxAGE 10p13 rs11258628

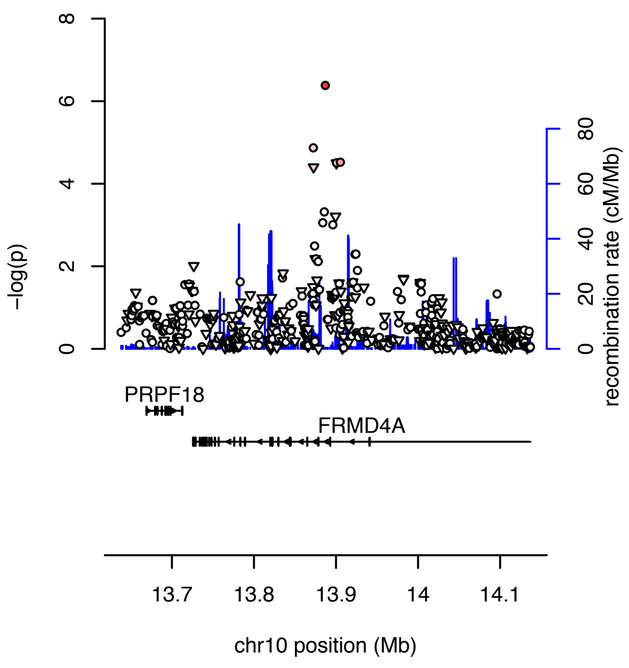

sbp_corExp_SNPxAGE 14q24.3 rs17104804

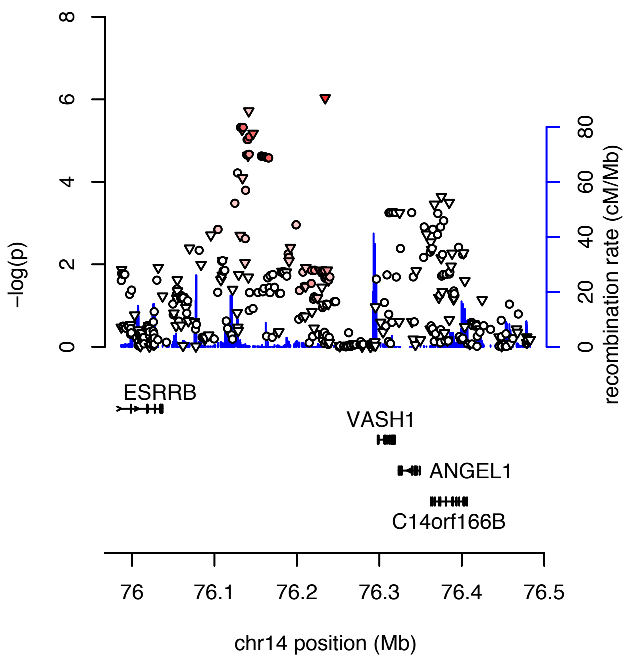

sbp_corExp_SNPxAGE 19p13.3 rs1046391

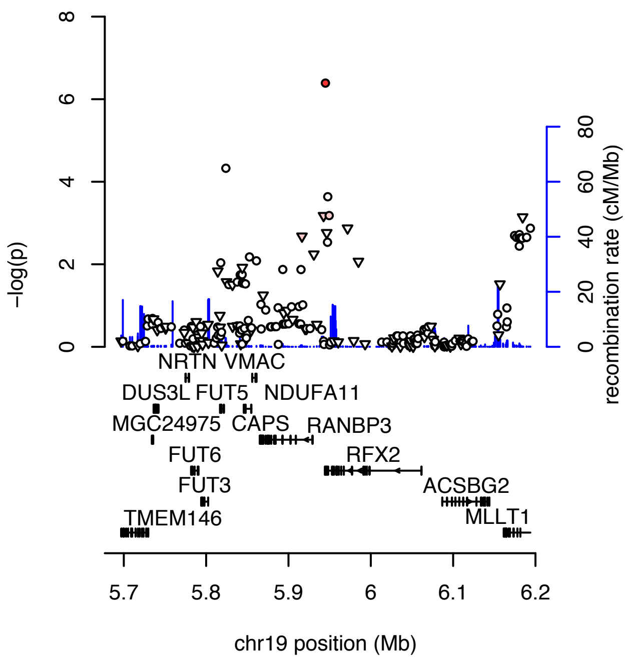

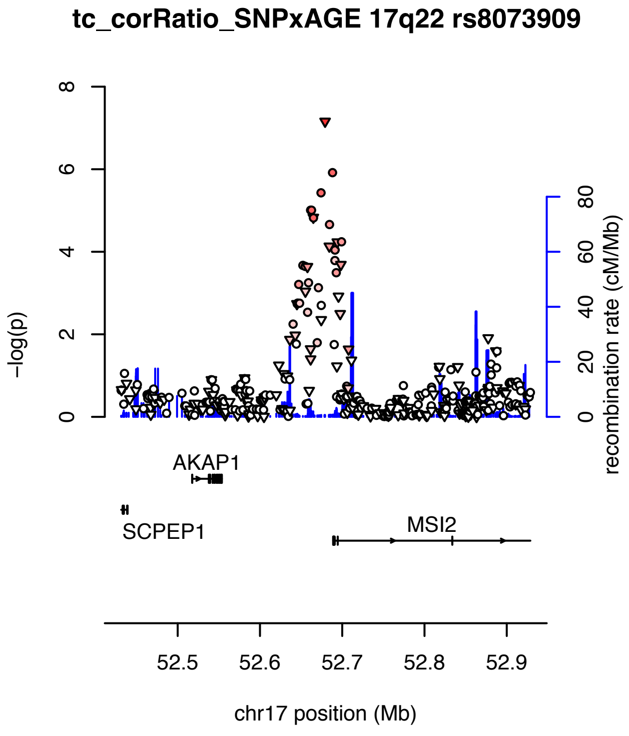

tc_corRatio_SNPxAGE 17q22 rs8073909
−log(p)
recombination rate (cM/Mb)
AKAP1
SCPEP1
MSI2
52.5
52.6
52.7
52.8
52.9
chr17 position (Mb)

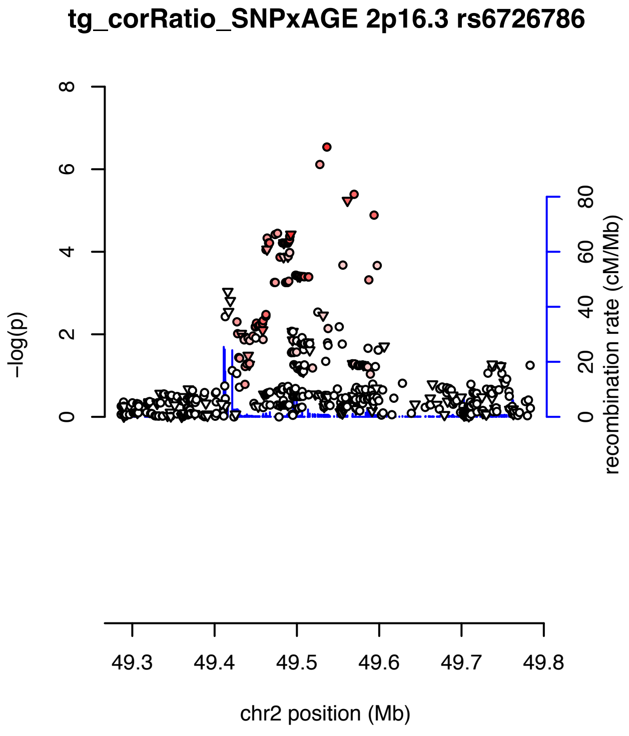

tg_corRatio_SNPxAGE 2p16.3 rs6726786
−log(p)
recombination rate (cM/Mb)
49.3
49.4
49.5
49.6
49.7
49.8
chr2 position (Mb)

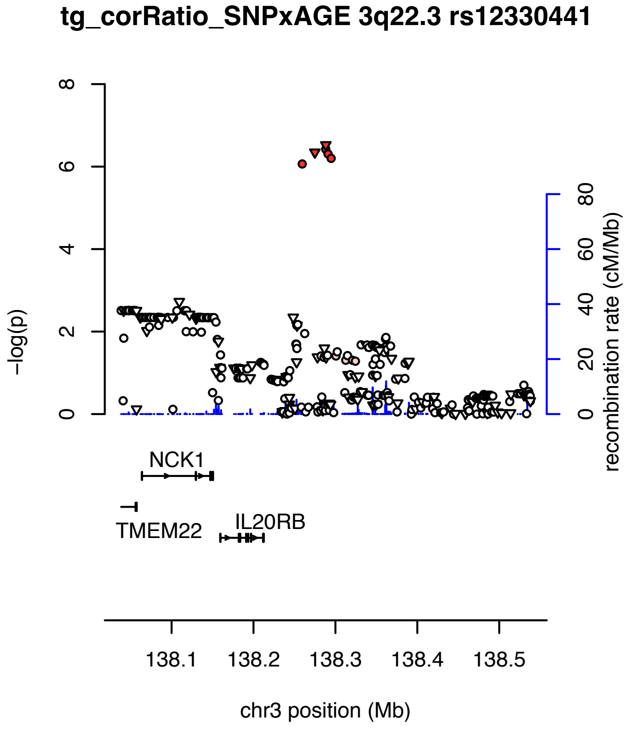

tg_corRatio_SNPxAGE 3q22.3 rs12330441
−log(p)
recombination rate (cM/Mb)
NCK1
TMEM22
IL20RB
138.1
138.2
138.3
138.4
138.5
chr3 position (Mb)

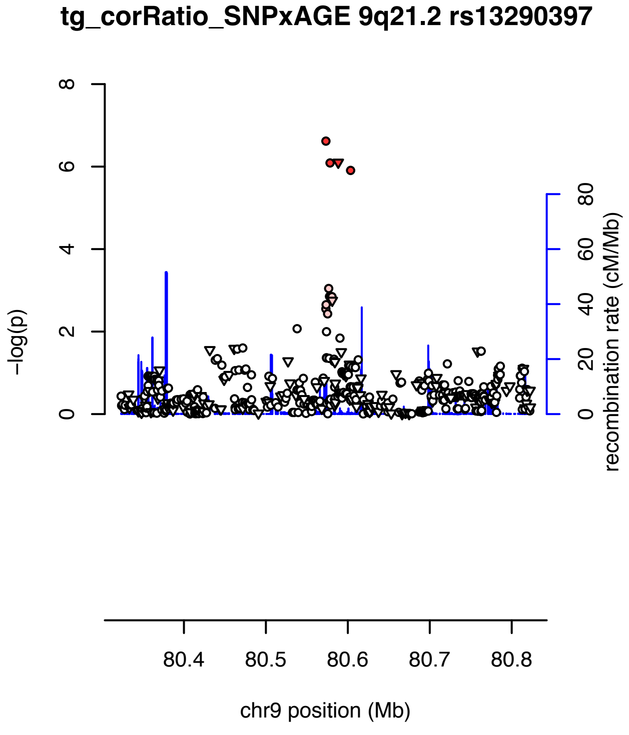

tg_corRatio_SNPxAGE 9q21.2 rs13290397
−log(p)
recombination rate (cM/Mb)
80.4
80.5
80.6
80.7
80.8
chr9 position (Mb)

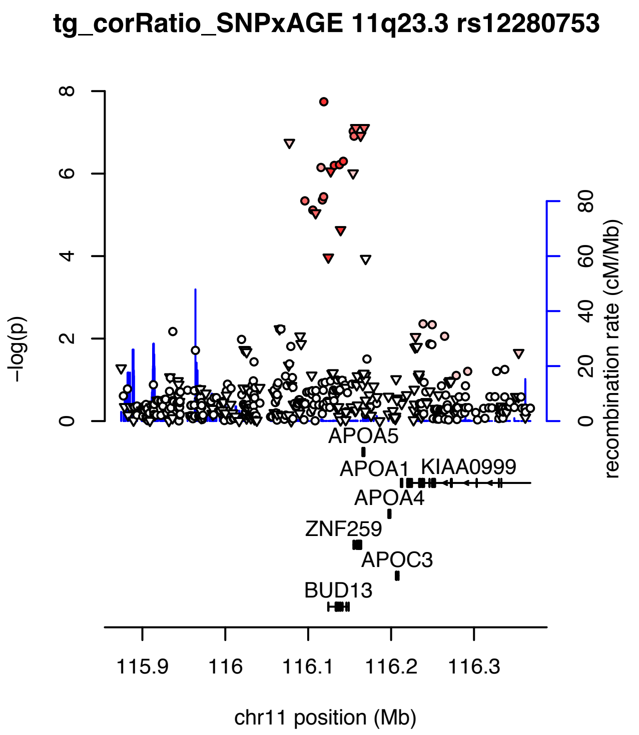
tg_corRatio_SNPxAGE 11q23.3 rs12280753
−log(p)
recombination rate (cM/Mb)
APOA5
APOA1
KIAA0999
APOA4
ZNF259
APOC3
BUD13
chr11 position (Mb)

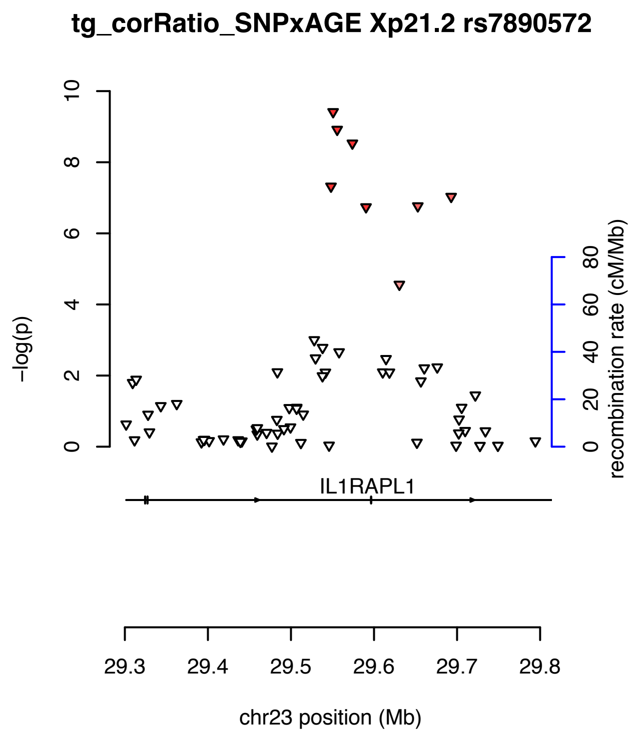
tg_corRatio_SNPxAGE Xp21.2 rs7890572
−log(p)
recombination rate (cM/Mb)
IL1RAPL1
chr23 position (Mb)

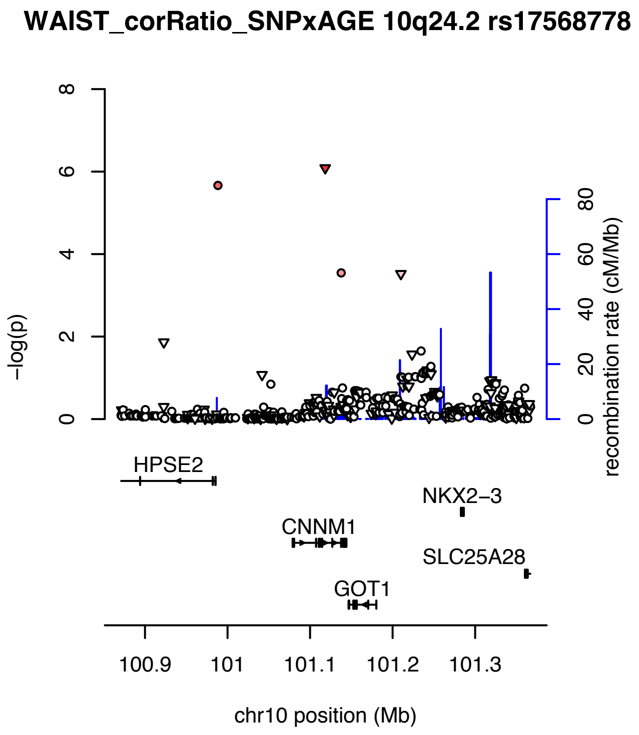
WAIST_corRatio_SNPxAGE 10q24.2 rs17568778
−log(p)
recombination rate (cM/Mb)
HPSE2
NKX2−3
CNNM1
SLC25A28
GOT1
chr10 position (Mb)

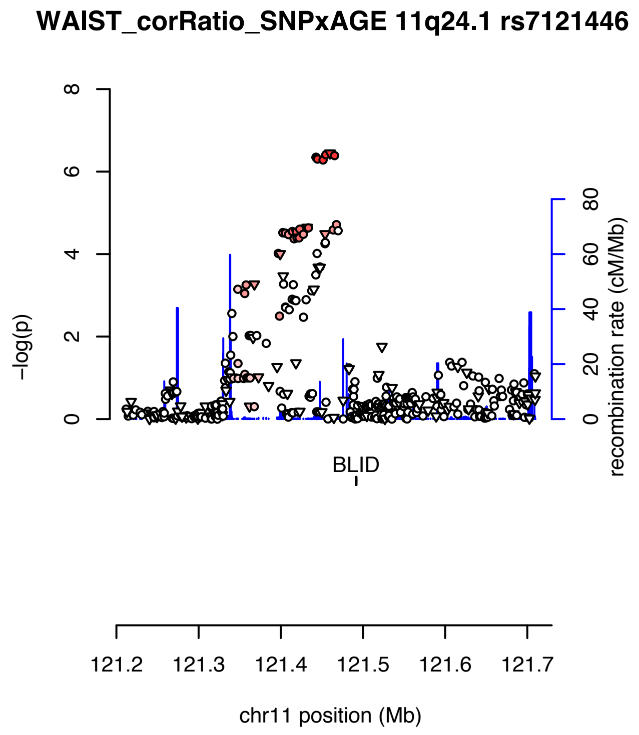
WAIST_corRatio_SNPxAGE 11q24.1 rs7121446
−log(p)
recombination rate (cM/Mb)
BLID
chr11 position (Mb)

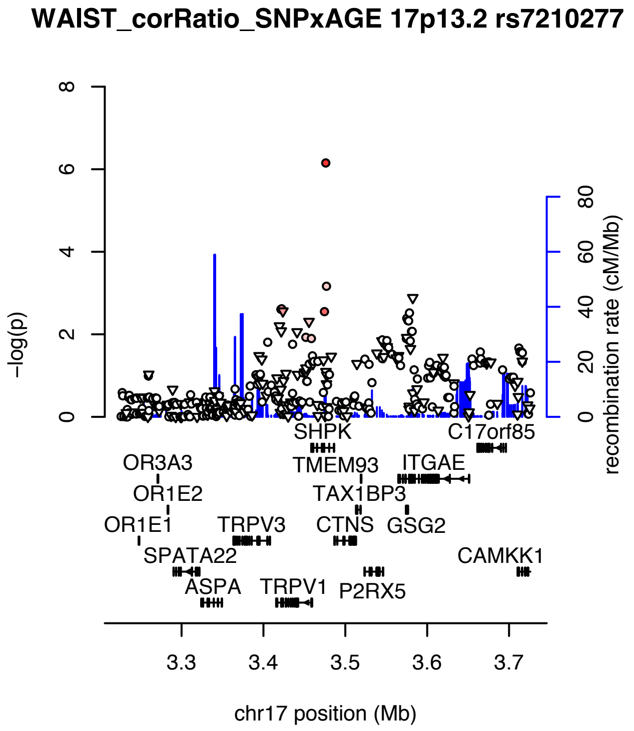
WAIST_corRatio_SNPxAGE 17p13.2 rs7210277
-log(p)
recombination rate (cM/Mb)
SHPK
C17orf85
OR3A3
TMEM93
ITGAE
OR1E2
TAX1BP3
OR1E1
TRPV3
CTNS
GSG2
SPATA22
CAMKK1
ASPA
TRPV1
P2RX5
3.3
3.4
3.5
3.6
3.7
chr17 position (Mb)

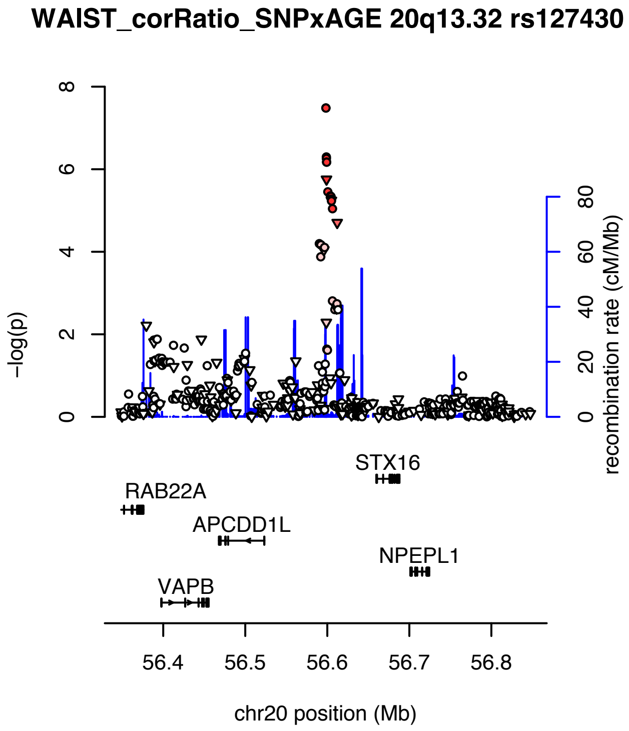
WAIST_corRatio_SNPxAGE 20q13.32 rs127430
-log(p)
recombination rate (cM/Mb)
STX16
RAB22A
APCDD1L
NPEPL1
VAPB
56.4
56.5
56.6
56.7
56.8
chr20 position (Mb)

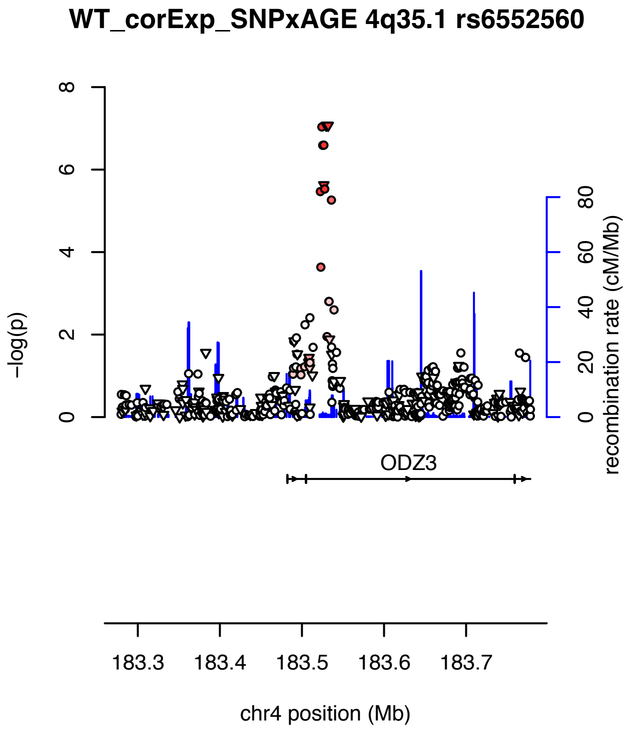
WT_corExp_SNPxAGE 4q35.1 rs6552560
-log(p)
recombination rate (cM/Mb)
ODZ3
183.3
183.4
183.5
183.6
183.7
chr4 position (Mb)
