# Supplementary material for: Longitudinal Genome-Wide Association of Cardiovascular Disease Risk Factors in the Bogalusa Heart Study
Source: PLoS Genet. 2010 Sep 9;6(9):e1001094. doi: 10.1371/journal.pgen.1001094 (PMC2936521; doi:10.1371/journal.pgen.1001094)

SNP effect Manhattan Plots

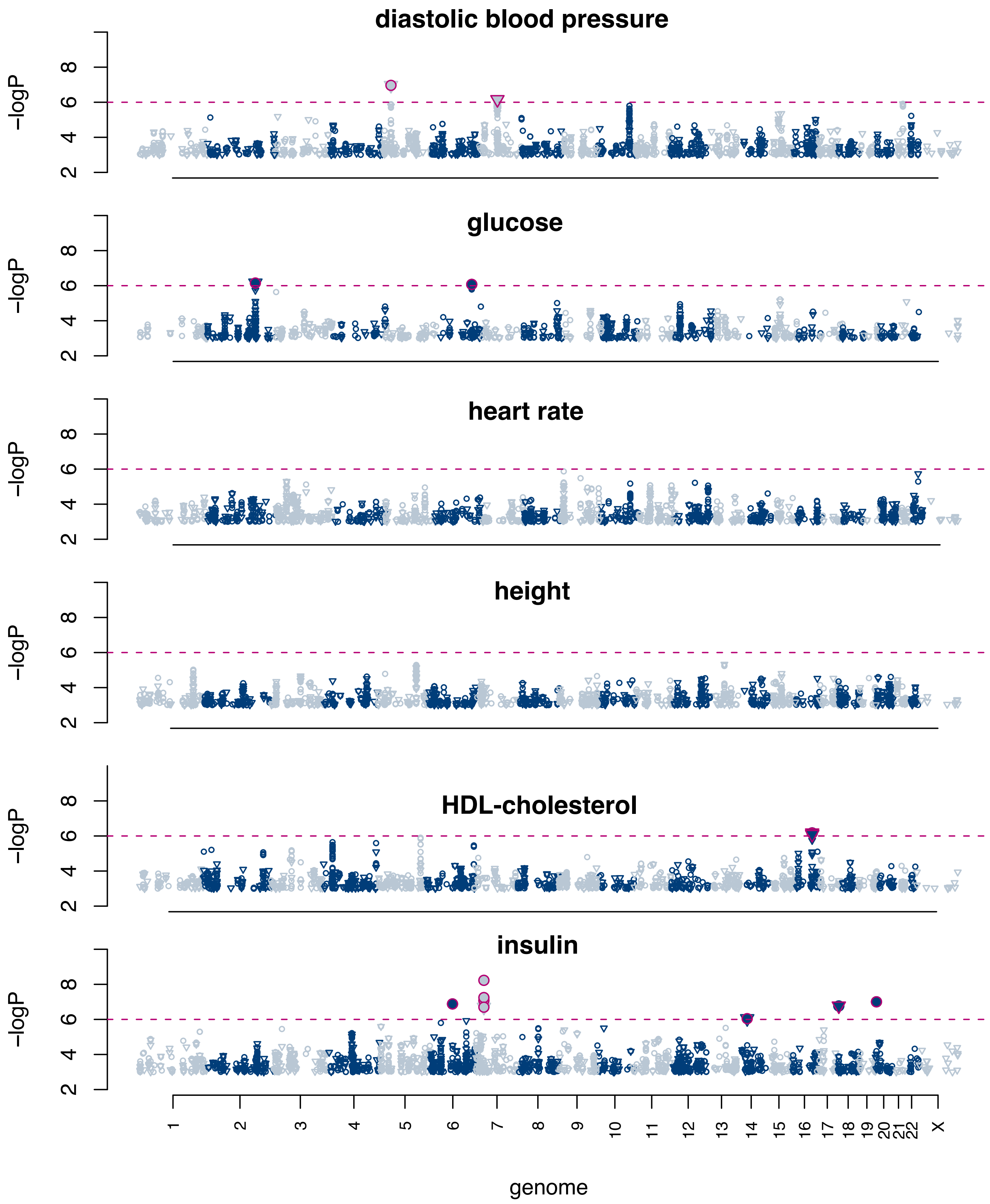

SNP effect Manhattan Plots

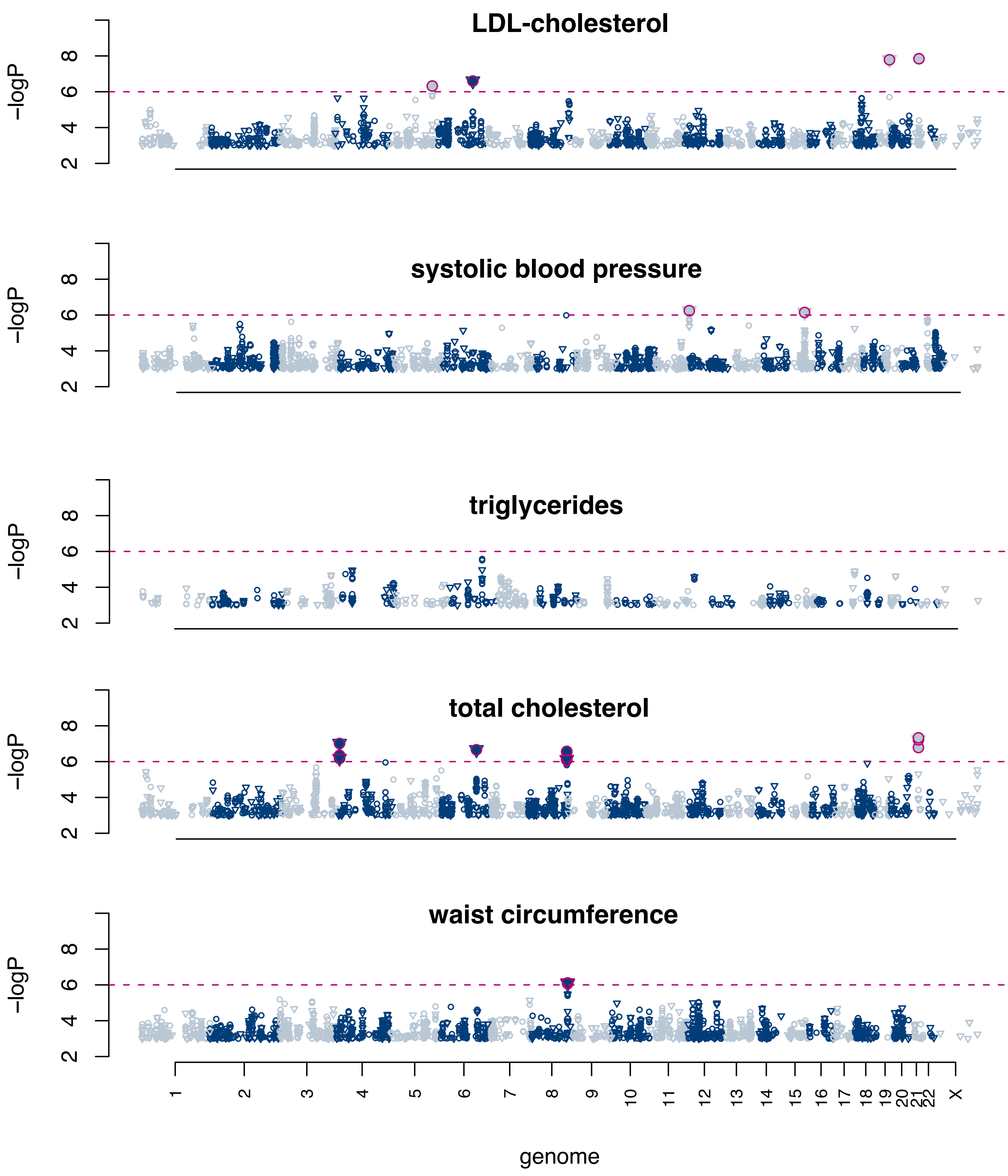

SNPxAGE effect Manhattan Plots

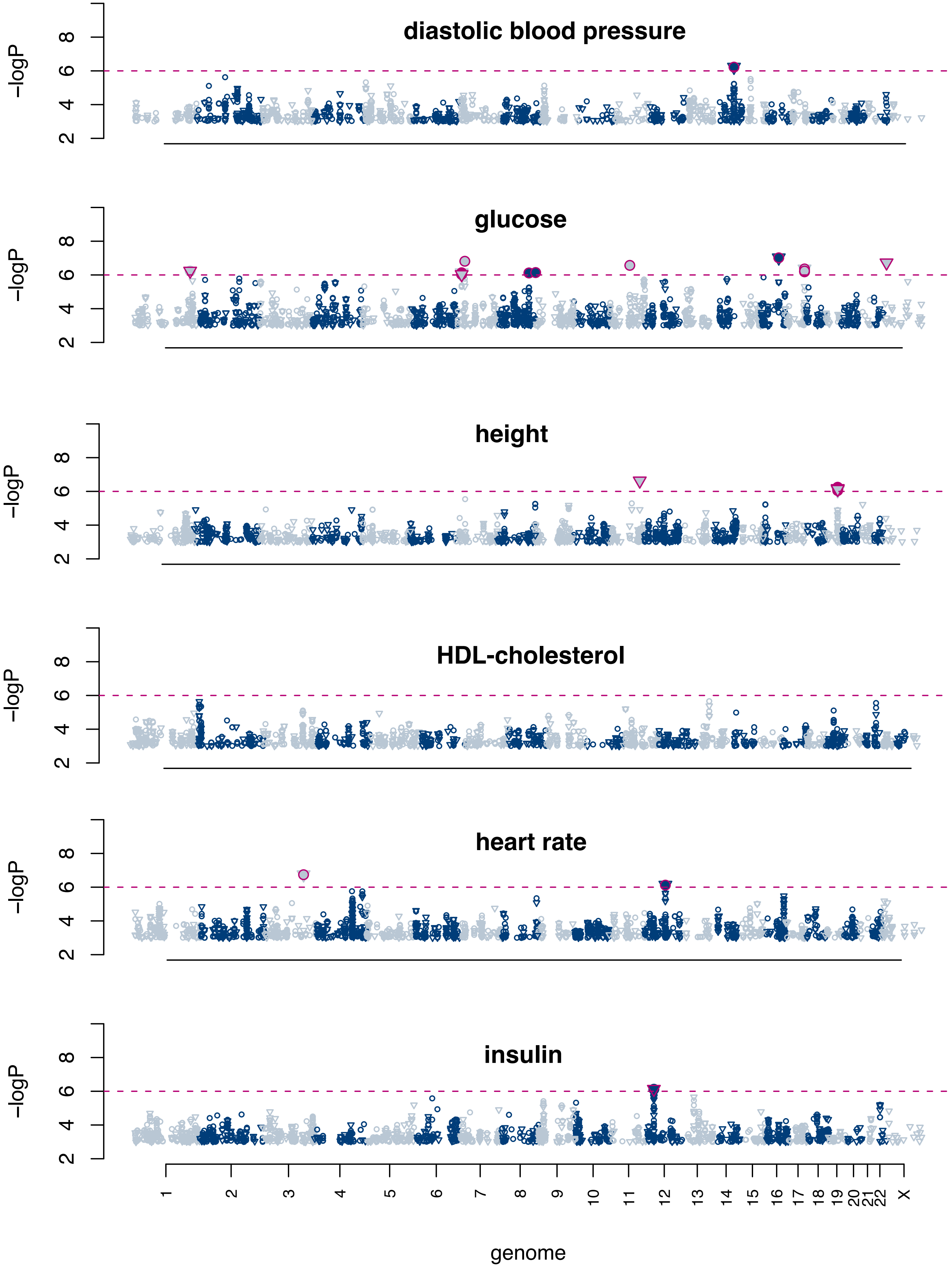

SNPxAGE effect Manhattan Plots

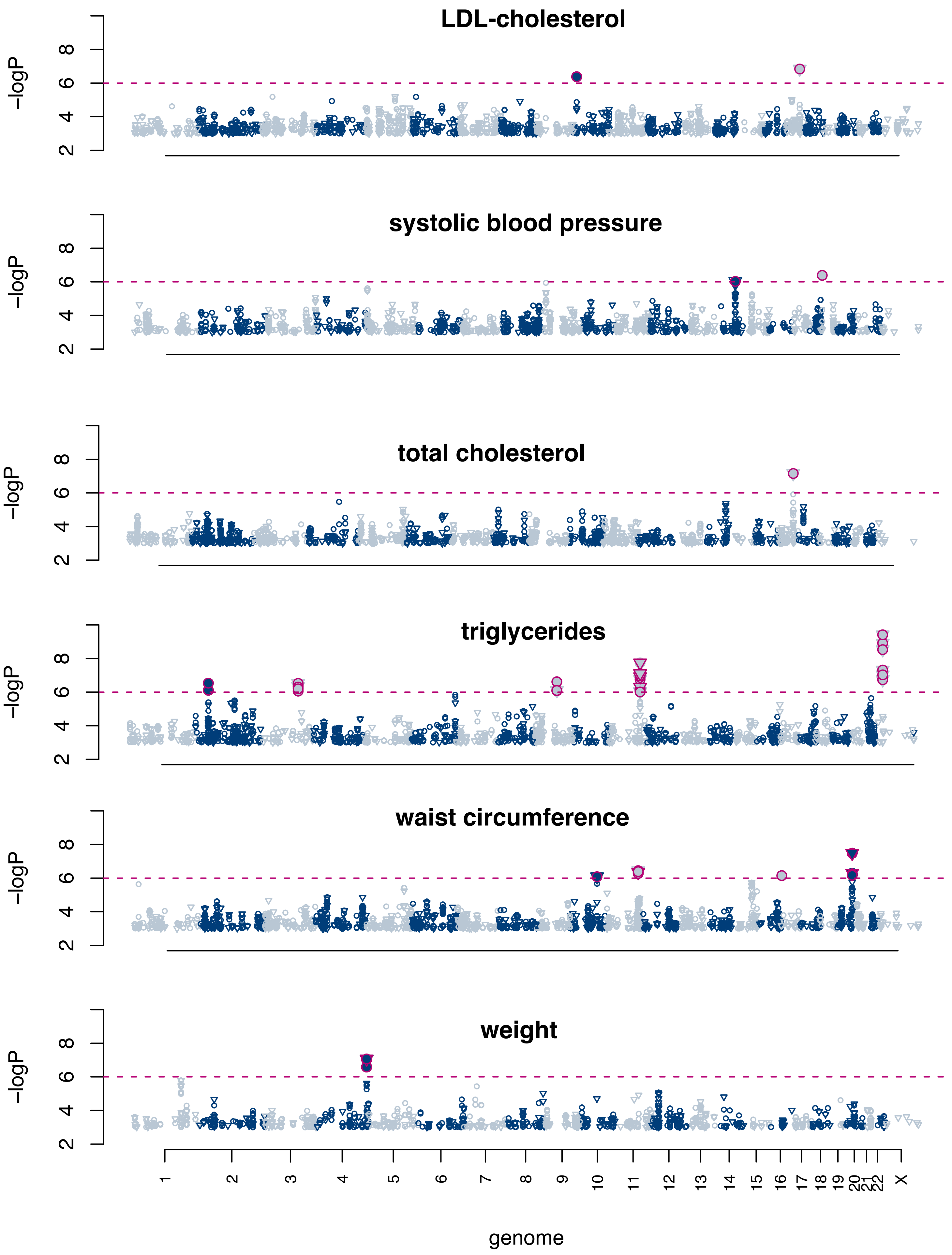

Supplement: Figure S2 — Manhattan Plots of GWAS results for each trait. Manhattan plots are shown for each SNP and SNPxAGE GWAS. Each point corresponds to an association with triangles indicating directly genotyped data and circles indicating imputed data. A horizontal line is plotted at P = 10−6 and SNPs above this point are outlined in pink. These SNPs occur in Tables 1, 2, and 3. Chromosomes are plotted in alternating blue and grey. P-values greater than 0.001 are not plotted. (8.19 MB PDF) [file pgen.1001094.s002.pdf]
