# Supplementary figures and images for: Longitudinal Genome-Wide Association of Cardiovascular Disease Risk Factors in the Bogalusa Heart Study
Source: PLoS Genet. 2010 Sep 9;6(9):e1001094. doi: 10.1371/journal.pgen.1001094 (PMC2936521; doi:10.1371/journal.pgen.1001094)

Figure S3

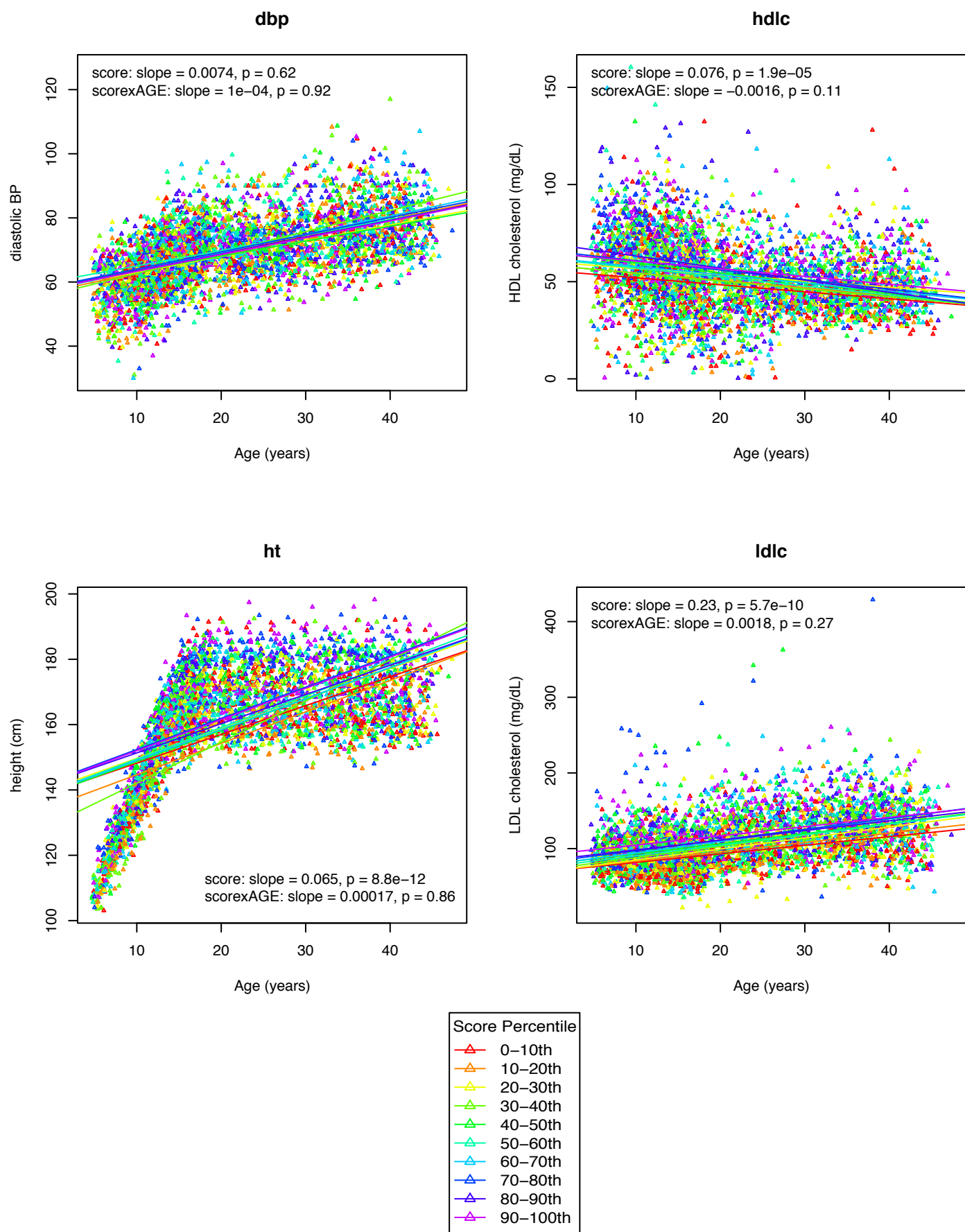

Figure S3

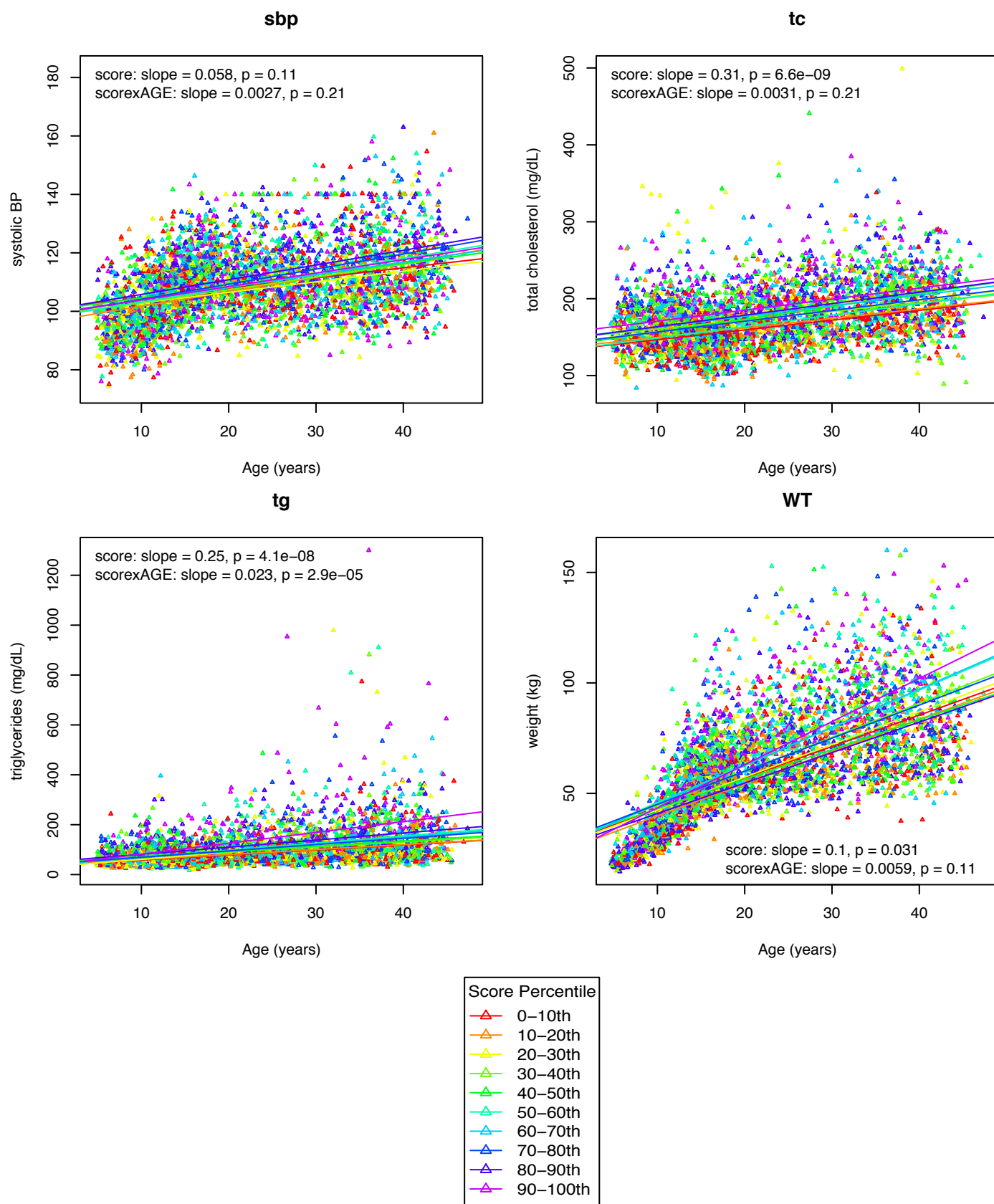

Supplement: Figure S3 — Longitudinal profiles of cumulative score from previously identified SNPs. Individuals were scored based on the effect size of each previously identified marker as in Figure 1B. Individuals are grouped and color-coded based on the decile of their score. Linear lines were calculated using linear regression with all points from all individuals in a given decile. (1.26 MB PDF) [file pgen.1001094.s003.pdf]

# Exam dates for 525 EA individuals in BHS

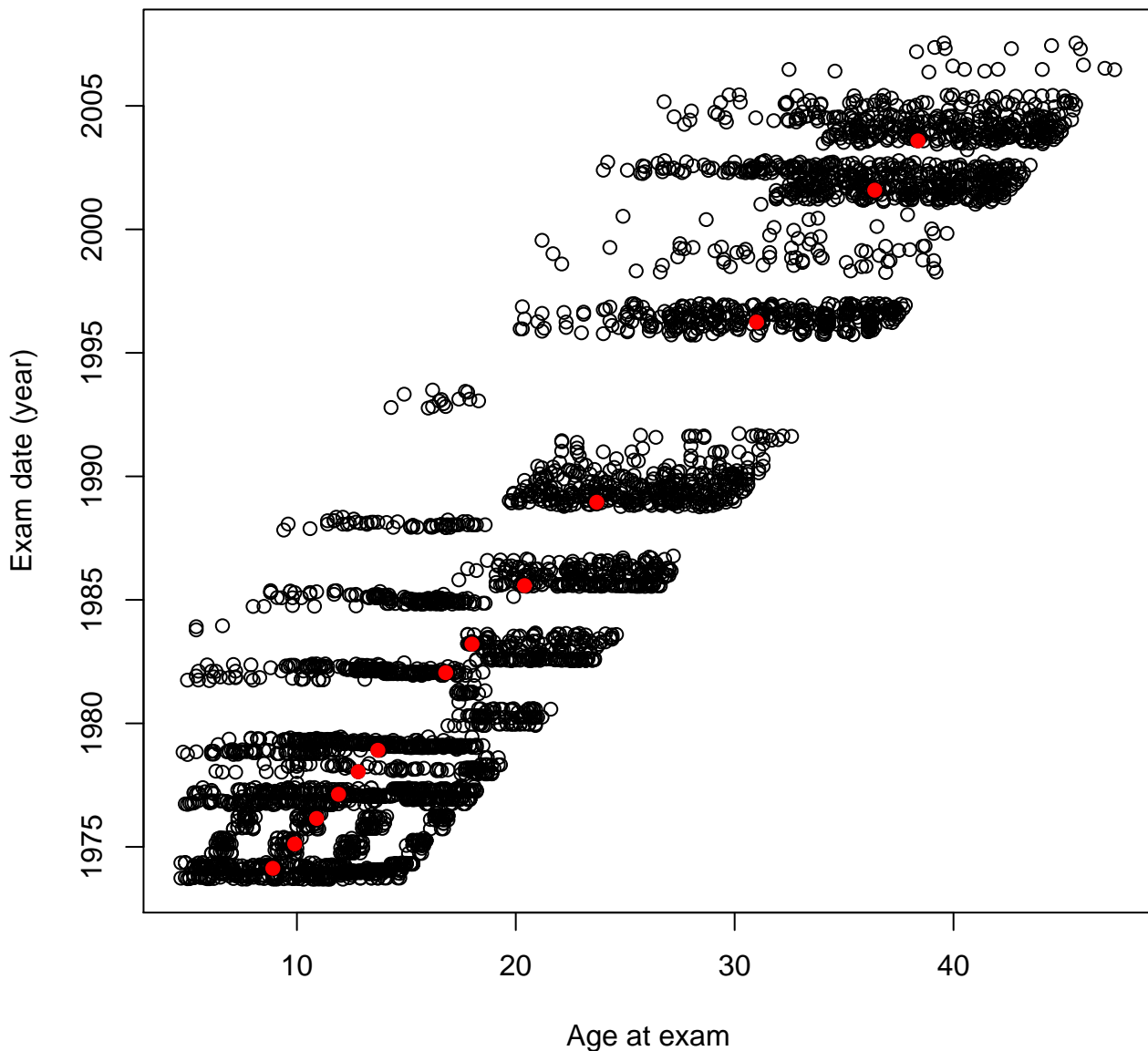

Supplement: Figure S4 — Age at measurement in the BHS. All exam dates that were included in the study are plotted as a function of the age of participant at the exam date. Individuals had between 4–13 measurements. A single individual is highlighted in red. (0.27 MB PDF) [file pgen.1001094.s004.pdf]
